# Synergistic Differential DNA Demethylation Activity of *Danshensu* (*Salvia miltiorrhiza*) Associated with Different Probiotics in Nonalcoholic Fatty Liver Disease

**DOI:** 10.3390/biomedicines12020279

**Published:** 2024-01-25

**Authors:** Amr Hassan, Patrícia Rijo, Tamer M. M. Abuamara, Lashin Saad Ali Lashin, Sherif A. Kamar, Gabrielle Bangay, Majid Mohammed Al-Sawahli, Marina K. Fouad, Mohammad A. Zoair, Tamer I. Abdalrhman, Dalia Elebeedy, Ibrahim A. Ibrahim, Aly F. Mohamed, Ahmed I. Abd El Maksoud

**Affiliations:** 1 Department of Bioinformatics, Genetic Engineering and Biotechnology Research Institute (GEBRI), University of Sadat City, Sadat 32897, Egypt; 2 CBIOS—Lusófona University’s Research Center for Biosciences and Health Technologies, 1749-024 Lisbon, Portugal; gabrielle.bangay@ulusofona.pt; 3 Instituto de Investigação do Medicamento (iMed.ULisboa), Faculdade de Farmácia, Universidade de Lisboa, 1649-003 Lisbon, Portugal; 4 Department of Basic Medical Science, Faculty of Dentistry, Al-Ahliyya Amman University, Amman 19111, Jordan; t.abuamara@ammanu.edu.jo (T.M.M.A.); l.lashin@ammanu.edu.jo (L.S.A.L.); s.qamar@ammanu.edu.jo (S.A.K.); 5 Department of Histology, Faculty of Medicine, Al-Azhar University, Cairo 11884, Egypt; 6 Department of Medical Physiology, Faculty of Medicine, Mansoura University, Mansoura 35516, Egypt; 7 Department of Anatomy and Embryology, Faculty of Medicine, Ain Shams University, Cairo 11566, Egypt; 8 Universidad de Alcalá de Henares. Facultad de Farmacia, Departamento de Ciencias Biomédicas (Área de Farmacología; Nuevos agentes antitumorales, Acción tóxica sobre células leucémicas), Ctra. Madrid-Barcelona km. 33,600, 28805 Alcalá de Henares, Madrid, España; 9 Department of Pharmaceutics, College of Pharmacy, The Islamic University, Najaf 54001, Iraq; majidalsawahli@gmail.com; 10 Department of Pharmaceutical Technology, Faculty of Pharmacy, Kafr Elsheikh University, Kafr Elsheikh 33516, Egypt; 11 College of Biotechnology, Misr University of Science and Technology, Giza 12573, Egypt; marinaaziz57@gmail.com (M.K.F.); daliaebeedy@hotmail.com (D.E.); aiaky79@gmail.com (A.I.A.E.M.); 12 Department of Physiology, Faculty of Medicine, Al-Azhar University, Cairo 11884, Egypt; drmohammads74@azhar.edu.eg; 13 Department of Histology, Faculty of Medicine, Al-Azhar University, Assiut 71524, Egypt; tamer23101982@gmail.com; 14 Department of Plant Biotechnology, Genetic Engineering and Biotechnology Research Institute (GEBRI), University of Sadat City, Sadat 32897, Egypt; brahim.ibrahim@gebri.usc.edu.eg; 15 Holding Company for Vaccine and Sera Production (VACSERA), Giza 22311, Egypt; fahmy.aly@gmail.com; 16 Department of Industrial Biotechnology, Genetic Engineering and Biotechnology Research Institute (GEBRI), University of Sadat City, Sadat 32897, Egypt

**Keywords:** *Salvia miltiorrhiza*, *Danshensu*, DNA demethylation, probiotics, nonalcoholic fatty liver disease, in silico, molecular docking, ADMET, hepatic

## Abstract

Nonalcoholic fatty liver disease (NAFLD) is a major hepatic disorder occurring in non-alcohol-drinking individuals. Salvianic acid A or *Danshensu* (DSS, 3-(3, 4-dihydroxyphenyl)-(2*R*)-lactic acid), derived from the root of *Danshen* (*Salvia miltiorrhiza*), has demonstrated heart and liver protective properties. In this work, we investigated the antioxidant activity and hepatoprotective activity of *Danshensu* alone and in combination with different agents, such as probiotic bacteria (*Lactobacillus casei* and *Lactobacillus acidophilus*), against several assays. The inhibition mechanism of the methylation gene biomarkers, such as DNMT-1, MS, STAT-3, and TET-1, against DSS was evaluated by molecular docking and RT-PCR techniques. The physicochemical and pharmacokinetic ADMET properties of DSS were determined by SwissADME and pkCSM. The results indicated that all lipid blood test profiles, including cholesterol (TC), triglycerides (TG), low-density lipoprotein cholesterol (LDL-C), and high-density lipoprotein cholesterol (HDL-C), were reduced after the oral administration of *Danshensu* combined with probiotics (*L. casei* and *L. acidophilus*) that demonstrated good, efficient free radical scavenging activity, measured using anti-oxidant assays. ADMET and drug-likeness properties certify that the DSS could be utilized as a feasible drug since DSS showed satisfactory physicochemical and pharmacokinetic ADMET properties.

## 1. Introduction

**Figure 1:**
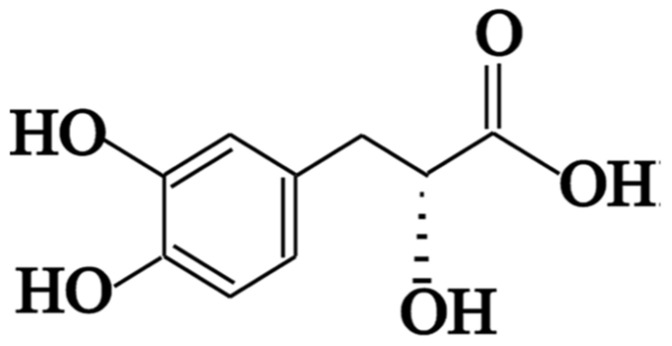
Chemical structure of 3-(3, 4-dihydroxyphenyl)-(2*R*) lactic acid, known as salvianic acid A or *Danshensu* (DSS), isolated from *Danshen* (*Salvia miltiorrhiza*).

Nonalcoholic fatty liver disease (NAFLD) pathogenesis is influenced by lifestyle, diet, and genetics [1,2]; however, it is dominated by elevated central adiposity [3]. NAFLD is influenced by many conditions, such as diet, genetics, and lifestyle, but the most important condition that affects NAFLD is high central adiposity [3]. Type 2 diabetes mellitus and obesity diseases are prevalent in those with NAFLD [4]. Epigenetic processes are related to different conditions, like the environment, genetics, and metabolic disease risk [5]. DNA methylation plays a central role in the epigenetic process due to its ability to change multigenetic processes, such as transcription and development [6,7]. The DNA methylation process is a transformation of a methyl group onto the C5 position of 5′-CpG-3′ dinucleotides to form 5-methylcytosine (5mC) through a specific enzyme, DNA methyltransferase (DNMT), with S-adenosyl methionine (SAM) as the active methyl donor [8]. The equilibrium between DNA methyltransferases and demethylases is vital for genomic methylation homeostasis. The deficiency in genomic methylation homeostasis leads to different diseases, such as cancer. The ten-eleven translocation 1 (TET-1) has a crucial function in the demethylation process through catalyzation because it hydroxylation from 5mC to 5hmC [9,10]. Also, DNMTs catalyze DNA methylation [6]. STAT 3-mediated microRNA (miRNA) expression is emerging as an epigenetic mechanism for driving hepatic oncogenesis [11]. Methionine synthase (MS), as methyl groups, was detected in liver tissue [12]. Currently, antihyperlipidemic medicine includes five main classes, which involve the following statins: fibric acid derivatives, bile acid binding resins, and nicotinic acid derivatives. In addition, some drugs inhibit cholesterol absorption [13]. Medicinal plants are rich sources of antioxidant, antibacterial, anti-inflammatory, and antitumor compounds [14]. Researchers are interested in medicinal plants’ antioxidant properties because of their superior effectiveness, safety, and consumer acceptability [15]. Many plants have anti-hyperlipidemia activity, such as *Salvia miltiorrhiza* (also known as *Danshen*), *Glycyrrhiza glabra*, and *Moringa oleifera* [16]. *Danshensu* is well known since salvianic acid A is extracted from it. Salvianic acid A (DSS) (Figure 1) is a purified component with a defined structure [17]. Previous studies focused on the activity of DSS as an anti-inflammatory agent, as well as its protective effects on the heart, liver, and kidney [18]. STAT 3 is only transiently activated in the liver under physiological conditions, due to the tight control of downregulation [19]. Probiotics are live microorganisms that have beneficial effects on the host when administered in appropriate amounts [20]. Lactic acid bacteria (LAB), which are generally regarded as safe (GRAS) bacteria, are more common due to their resistance to bile toxicity as well as their ability to tolerate gastric acidity in the intestine [21]. *Lactobacillus* probiotic strains are reported to control hyperglycemia, lactose intolerance, and insulin [22,23]. Previous studies demonstrated that both *L. acidophilus* and *L. casei* are capable of improving lipid profiles [19], fatty liver grade, and inflammatory and anti-oxidative status [24]. Also, *L. casei* can be reduced to firmicutes [25,26]. In our study, we studied the effect of *Danshensu* associated with different probiotics as an antioxidant activity and hepatoprotective agent. We also examined the activity of DSS as a DNA demethylation agent in vivo and in silico using RT-PCR and molecular docking.

## 2. Materials and Methods

### 2.1. In Vitro Study of Danshensu Extracts

Phytochemical Analysis.

### 2.2. Total Polyphenol Content (TPC)

The total polyphenol content (TPC) of *Danshensu* extracts was evaluated using method outlined by Yawadio Nsimba et al. [27]. In brief, 0.5 mL of *Danshensu* extracts was mixed with diluted Folin-Ciocalteu reagent in a ratio of 1:10 for 5 min at room temperature, followed by the addition of sodium carbonate solution (2 mL, 7.5% *w*/*v*). The mixture was left to stand for half an hour at room temperature (RT), and then absorbance was determined at 765 nm. The quantitative value of TPC was determined as milligrams of gallic acid equivalents (GAE) per 100 g (mg of GAE g^−1^ dw).

### 2.3. Determination of Total Flavonoids (TFs)

TF was measured using the aluminum chloride colorimetric assay described by Zhisen et al. [28]. Briefly, 0.5 mL of *Danshensu* extracts was mixed with 150 μL of 5% sodium nitrate and 2.5 mL of distilled water for 5 min; then, 0.3 mL of 10% AlCl_3_ was added. At 6 min, 1 mL of 0.001 M NaOH and 0.55 mL of distilled water were added to the mixture and left at RT for 15 min. The absorbance was determined at 510 nm. The content of TF was expressed as mg of quercetin equivalent (QE) per g^−1^.

### 2.4. Measurement of Antioxidant Properties

The DPPH (1, 1-diphenyl-2-picrylhydrazyl) scavenging effects of *Danshensu* were evaluated using a standard method [29]. A freshly prepared solution of DPPH in methanol (6 × 10^−5^ M) was used for the UV measurements. The samples of different concentrations (10–80 μg/mL) were added to the DPPH solution in a 1:1 ratio, followed by vortexing. Then, the assay took place in a dark room at room temperature. Ascorbic acid and trolox served as standards. The inhibition percentage of DPPH radical scavenging activity was calculated using the following equation:

*Inhibition (%)* = [(*A*_0_ − *A*)/*A*_0_] × 100

where *A*_0_ is the absorbance of DPPH in the absence of the sample, and *A* is the absorbance of DPPH during the existence of the test material. The IC_50_ values were evaluated using a graph between the inhibition percent and the sample concentration.

### 2.5. Total Reduction Capability

The total reduction capability of the examined materials was measured according to the method of Kumari et al. (2016) [30]. Briefly, the mixture of 2.5 mL of 0.2 M phosphate buffer (pH 6.6) and 2.5 mL of 1% potassium ferric cyanide was added to 1 mL of Danshensu in various concentrations (10–160 μg/mL), followed by gentle mixing. This was followed by incubating the mixture inside a water bath at 50 °C, then adding 2.5 mL of stopping reaction solution (10% TCA) and centrifuging the mixture at 4000 rpm for 10 min. Then, we pulled 2.5 mL from the top layer and transferred it to a new tube, completing it with 2.5 mL of distilled water and 0.5 mL of 0.1% FeCl_3_.6H_2_O. This was mixed for 5 min. The absorbance was measured at 700 nm against the blank. Ascorbic acid and trolox were assigned as standards.

### 2.6. ABTS Radical Cation Decolorization Assay

The ABTS+ radical cation scavenging assay is one of the quantitative methods to measure and evaluate the antioxidant activity of *Danshensu*. In brief, 7 mM of 2 and 20-azino-bis (3-ethylbenzothiazoline-6-sulfonic acid) diammonium salt (ABTS) was mixed with 2.45 mM potassium persulfate and let stand at room temperature in the dark. After that, 3 mL of ABTSC solution was gently mixed with 0.2 mL of *Danshensu* extracts at different concentrations (10–160 μg/mL). We utilized ascorbic acid and trolox as standards. The mixture was left to stand at RT for 6 min [31]. The inhibition percentage was determined by using the following equation:

*% Inhibition* = *Optical Density of control* − *Optical Density of test material* = *Optical Density of control* × 100

### 2.7. Hydrogen Peroxide (H_2_O_2_) Radical Scavenging Activity

The radical scavenging activity of the extracts against H_2_O_2_ was measured using the method of Ruchetal (1989) [32]. Briefly, water extracts of DSS (10–160 μg/mL) were mixed with 0.6 mL of hydrogen peroxide (40 mM) in the prepared phosphate buffer (pH 7.4). Then, the mixture was incubated at RT for 10 min. After that, the mixture was scaled at 230 nm against the blank solution, and both ascorbic acid and trolox were used as standards. The inhibition percentage was estimated by applying the following formula:

*Percentage (%) of inhibition* = (*A*_1_ − *A*_2_)/*A*_1_ × 100

where *A*_1_ is the absorbance of the hydrogen peroxide and *A*_2_ is the absorbance of the reaction mixture with *Danshensu extract*.

### 2.8. Nitric Oxide (NO) Radical Scavenging Assay

Nitric oxide (NO) radical scavenging assay was determined and evaluated by the Sreejayan and Rao (1997) method [33]. NO radicals were generated from sodium nitroprusside solution and 1 mL of Danshensu extracts at various concentrations (10–160 μg/mL). The mixture was incubated at 25 °C for 150 min, followed by mixing with 1.0 mL of pre-prepared Griess reagent. Ascorbic acid and trolox were assigned as standards.

The inhibition was measured by applying the following equation:

*% Inhibition of NO radical* = [*A*_0_ − *A*_1_]/*A*_0_ × 100

where *A*_0_ is the absorbance before the reaction and *A*_1_ is the absorbance after the reaction has taken place with Griess reagent. The decreasing absorbance indicates a high NO scavenging activity.

### 2.9. In Vitro Lipid Peroxidation (LPO) Assay

#### 2.9.1. Preparation of Rat Liver Homogenate

Male Wistar Albino rats, weighing approximately 170–200 g, were sacrificed after being anesthetized with a standard anesthetizer, sodium pentobarbitone (35 mg/kg), followed by the excision of one lobe of the liver, washing it with a saline solution. Hepatic homogenate tissues were prepared by homogenizing 1 g of hepatic tissue and 05 M ice-cold phosphate buffer (pH-7.5) in a ratio of 1:10 in a Teflon homogenizer. Hepatic homogenate tissues were used to determine thiobarbituric acid-reactive substances (TBARSs).

#### 2.9.2. TBARS Assay

Lipid peroxidation is classified as an oxidative stress marker that is generated in liver tissue through the induction of Fe^2+^ ascorbate pathway. The lipid peroxidation (LPO) assay was carried out using the common method of Ohkawa et al. (1979) [34]. Hepatic homogenate (0.25 mL) was mixed with 0.1 mL of Tris HCL buffer (pH 7.2), 0.05 mL of 0.1 mM ascorbic acid, 0.05 mL of 4 mM FeCl_2_ solution, and 0.05 mL of the *Danshensu* extracts at various concentrations (10–160 μg/mL). The mixture was incubated at 37 °C for 1 h, and 1.5 mL of 0.8% (*w*/*v*) 2-thiobarbituric acid, 1.5 mL of 20% acetic acid, and 0.2 mL of 8.1% (*w*/*v*) sodium dodecyl sulfate were added to the reaction mixture. The mixture was made up to 4.0 mL with distilled water and heated at 95 °C for 60 min. After cooling with tap water, 1.0 mL of distilled water and 5.0 mL of a mixture of n-butanol and pyridine (15:1, *v*/*v*) were added. The mixture was shaken vigorously. The absorbance was measured at 532 nm in a spectrophotometer (Beckman, UK). Ascorbic acid and trolox are assigned as standards.

### 2.10. In Vivo Study

#### 2.10.1. Animals and Experimental Design

We carried out the experimental technique after the Animal Protocols Evaluation Committee’s affirmative decision, according to the Ethics Committee of the faculty of veterinary medicine at the University of Sadat City, Egypt (IACUC, VUSC-023-1-22) [35,36]. Thirty male Wistar rats were used in this study. Rats weighing 170–200 g were purchased from VACSERA’s animal home in Giza, Egypt, at the age of 8–10 weeks. The rats were kept in a controlled environment with food and water, including a consistent room temperature of 22–24 °C and a 12 h light–12 h dark cycle. The rats were fed a dry chow diet and had access to water at all times. For the experiment, the rats were placed into six groups, each with five rats.

#### 2.10.2. Acute Toxicity Study

Acute toxicity study was carried out as per the Ethics Committee of the faculty of veterinary medicine at the University of Sadat City guidelines (acute toxic class method). Waster rats (n = 5) of either gender were selected by random sampling for the acute toxicity study. The Wistar Albino rats did not feed overnight, but a single water *ad libitum* dose of 5 mg/kg body weight (b.w.) was administered under monitoring and followed for 14 days. The following protocol was used: (i) if mortality was noticed in two out of three rats, then the dose administered was assigned as a toxic dose; (ii) if mortality took place in one out of three, the dose was repeated to emphasize the toxicity; and (iii) if there was no mortality at all, the dose specificity was increased until it reached the high maximum of 3000 mg/kg b.w.

#### 2.10.3. Induction of Hyperlipidemia

According to Khanna et al., all rats weighing between 100 and 110 g were divided into 6 groups (A-F) and fed 12 g of diet each day. All groups except group A were given intragastric administration each day for 28 days while being fed a high-cholesterol diet (HCD) that included whole wheat (62.5 g), yellow corn (37.5 g), barley, and one tablet of vitamin B12. After giving the rats a week to acclimate, they were divided into six groups, each with six rats: Group A—normal diet and water (control); Group B—normal diet plus cholesterol (25 mg/kgbw/day); Group C—normal diet + cholesterol (25 mg/kg b.w./day) + fenofibrate (650 mg/kg b.w./day); Group D—normal diet + cholesterol (25 mg/kg b.w./day) and DSSE (50 mg/kg/day); Group E—normal diet + cholesterol (25 mg/kg b.w./day) + *L. acidophillius* mixture of 2 × 10^8^ CFU/mL plus DSS (50 mg/kg/day); Group F—normal diet + cholesterol (25 mg/kg b.w./day) + *L. casei* mixture of 2 × 10^8^ CFU/mL plus DSS (50 mg/kg/day). All animals were fed for 28 days. Then, animals were sacrificed after fasting for 12 h, according to the Ethics Committee of the faculty of veterinary medicine at the University of Sadat City, Egypt (IACUC, VUSC-023-1-22) [37].

#### 2.10.4. Collection of the Body Organs (Heart, Liver, and Serum)

The animals were fasted for 12 h prior to being put under anesthesia, after receiving food for 28 days. By cervical dislocation, the anesthetized rats were sacrificed [37]. The heart, liver, and kidneys, among other complete animal organs, were all removed and weighed. For the next tests, the liver was collected and kept at −80 °C. Additionally, blood was drawn using an intracardiac puncture, separated into serum using centrifugation, and then collected in heparinized tubes. The serum samples were then well mixed by being gently inverted 2–3 times and incubated at 4 °C for 2–3 h [34]. All bodily organs, including the liver, kidney, pancreas, and heart, were fixed in formalin and embedded in paraffin for histological studies.

#### 2.10.5. Preparation of Tissue Homogenate

Homogenization of 1 g of wet tissue in 10-times (*w*/*v*) 0.05 M ice-cold phosphate buffer (pH 7.4) in a Teflon homogenizer produced the animal’s liver, heart, and kidney tissue homogenate. The homogenate was then split into two parts: the first part was used to assess SOD using the supernatant of homogenate tissues after centrifugation at 15,000× *g* at 4 °C for 60 min, and the other part was used to assess GSH by mixing with 10% TCA in a ratio of 1:1 and centrifugation at 5000 rpm at 4 °C for 10 min, finally separating the supernatant for use in studying GSH.

### 2.11. Biochemical Analysis

#### 2.11.1. Blood Lipid Profile Analysis

All the blood lipid profiles were evaluated and calculated using Friedwald’s formula [38].

#### 2.11.2. Determination of HMG-CoA Reductase Activity

The HMG-CoA reductase was tested by using a commercial kit purchased form Sigma-Aldrich (St. Louis, MO, USA). The assay depends upon the oxidation reaction of NADPH through the catalytic process of the subunit of HMG reductase in the existence of the substrate HMG-CoA at 37 °C according to the manufacturer’s protocol [39].

#### 2.11.3. Determination of Hepatic and Fecal Lipids

Total lipids from the liver samples were determined by using a mixture of chloroform and methanol with a 2:1 ratio (*v*/*v*) [40]. Briefly, one hundred grams of liver tissues was homogenized in 500 μL of phosphate buffer (pH 7.4) and centrifuged at 9000× *g* for 10 min at 4 °C. Then, the supernatant was separated and added to 1 mL of the chloroform/methanol mixture. The mixture was sonicated for 30 min and allowed to stand for 1 h, followed by the addition of 1000 μL isopropanol and vortexing the samples before the analysis. The fecal samples were collected before the end of the experiment, and the chloroform/methanol method was used. Hence, cholesterol and triglycerides were measured using standard kits obtained from Thermo Fisher Scientific (Waltham, MA, USA).

#### 2.11.4. Measurement of Fecal Bile Acids

According to Zhu et al. (2008) [37], bile acids from feces were measured. Then, 48 h before the completion of the experiment, fecal samples were collected, dried, weighed, and frozen at −70 °C. The pollutants were then removed from 100 mg of feces using 1 mL of a chloroform/methanol (2:1 *v*/*v*) solution and 2 mL of KCl (3.7 g/L). The top layer was then removed from the sample, evaporated, and dissolved in 1 mL of 50% methanol after being centrifuged at 1500× *g* for 10 min. A 20 μL sample was combined with 30 μL of 3α- hydroxysteroid dehydrogenase (0.1 IU per sample), 250 μL of NAD (2 mmol/L) diluted in phosphate buffer (pH 10.5), and 30 min of incubation at room temperature [38].

#### 2.11.5. Evaluation of Tissue Markers of Oxidative Stress

According to Ohkawa et al., (1979), we determined thiobarbituric acid-reactive substances as indicators of lipid peroxidation in serum, heart, and liver tissues [34], while glutathione (GSH) was determined by Ellman (1959) [39] and superoxide dismutase (SOD) was determined by Marklund (1974) [40]. The nitrate oxide (NO) level was assayed using the method described by Green et al. (1982) [41].

### 2.12. Quantitative RT-PCR

Total RNA from the liver in RNA later solution was extracted using a Fast RNA Pro Green Kit according to the manufacturer’s instructions. Gene expression was measured using a two-step multiplex quantitative RT-PCR method. The nucleotide positions of the oligonucleotides are listed in Table 1. Real-time PCR was performed using a Bio-Rad DNA Engine (Bio-Rad Lab. Inc., Hercules, CA, USA) according to the manufacturer’s instructions. Reactions were performed in a 25 μL volume with forward primer, reverse primer, SYBR Green Real-time PCR Master Mix-Plus, template, and DEPC water. Then, we added 2 μL of template cDNA to the final volume of 20 μL of the reaction mixture. The procedures occurred as follows: enzyme activation for 10 min at 95 °C, then 40 cycles to denature for 15 s at 995 °C, an annealing step for 20 s at 555 °C, and an elongation step at 72 °C for 20 s [42].

### 2.13. In Silico Study

#### 2.13.1. Ligand Preparation

The 2D structure of DSS was obtained from the PubChem database (PubChem CID: 11600642) [43]. All the sketched 2D structures were transformed into 3D, and geometry was optimized by using Avogadro 1.2.0 software [44]. Geometry optimization was used to find the most stable conformers of all the molecules. The results were saved in a separate folder in PDB format.

#### 2.13.2. Receptor Preparation

The two-dimensional X-ray crystal structures of the following proteins were obtained from the RCSB Protein Data Bank (https://www.rcsb.org/) and uniport prepared as receptors for molecular docking: DNMT-1 (PDB ID: 7sfc, MS (PDB ID: 2o2k), STAT-3 (PDB ID: 6njs), and HMG (Uniport ID: P43256). The crystal structures were prepared by removing heteroatoms, water, and ions. Hydrogens were added and missing residues were built using AutoDock Tools 1.5.7. The prepared structures were saved as PDBQT files [45].

#### 2.13.3. Molecular Docking

The software AutoDock 1.5.2 was utilized to carry out all molecular docking calculations. The PDBQT file of receptors was prepared by the AutoDock protocol. The maximum number of energy evaluations (evals) and the genetic algorithm number (GA) were changed to 250 and 25,000,000, respectively. The default settings for every other option were retained. The active sites were defined by the docking grid’s dimensions [46].

#### 2.13.4. ADMET and Drug-Likeness Properties

The pharmacokinetics of DSS were investigated using the accessible websites SwissADME (http://www.swissadme.ch/) and pkCSM (https://biosig.lab.uq.edu.au/pkcsm/ accessed on 24 December 2023), which were used to study the ADMET and drug-likeness of the study compound. Interestingly, Lipinski’s rule of five (ROF) was evaluated to screen the opportunity of DSS to work as a standard drug [47,48,49].

### 2.14. Statistical Analysis

Using the SPSS 17 software packages (SPSS Inc., Chicago, IL, USA), the statistical analysis of each experiment was assessed. One-way ANOVA was used to assess the data, and *p* < 0.05 was considered to be a significant value. Each experiment was independently analyzed by three researchers. We also calculated mean values and standard deviations [50,51,52].

## 3. Results

### 3.1. Phytochemical Analysis

The quantitative analysis of *Danshensu* extracts summarized in Table 2 shows that the phytochemical analysis of both TPC and TF content was 111.9 mg GAE g^−1^ and 33.79 mg QE g^−1^, respectively.

### 3.2. In Vitro Antioxidant Activities of Danshensu

#### 3.2.1. DPPH Radical Scavenging Activity

**Figure 2:**
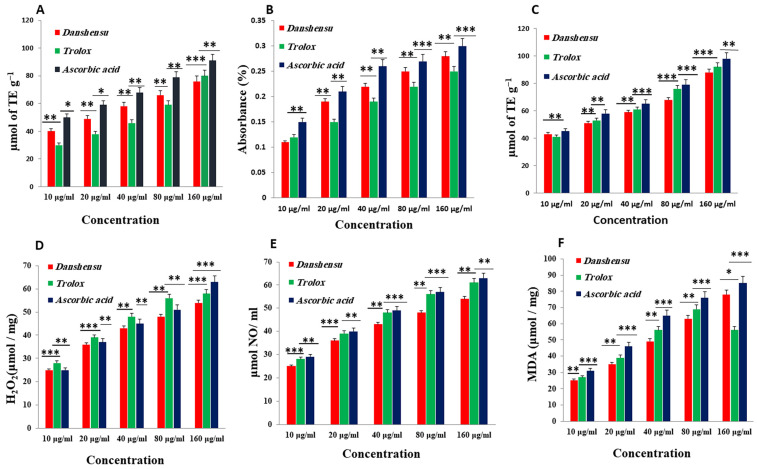
Antioxidant activity of *Danshensu* extract at various concentrations (10–160 μg/mL). (**A**) 1, 1-diphenyl-2-picrylhydrazyl (DPPH) activity. (**B**) Total reduction capability (TRC) (**C**) ABTS radical cation decolorization (ABTS) (**D**) hydrogen peroxide (H_2_O_2_). (**E**) Nitric oxide (NO) (**F**) lipid peroxidation (LPO) assay. Each point represents the mean ± SD (*n* = 3). ***, **, and * shows statistically significant differences at *p* < 0.001, *p* < 0.01, and *p* < 0.05.

**Figure 3:**
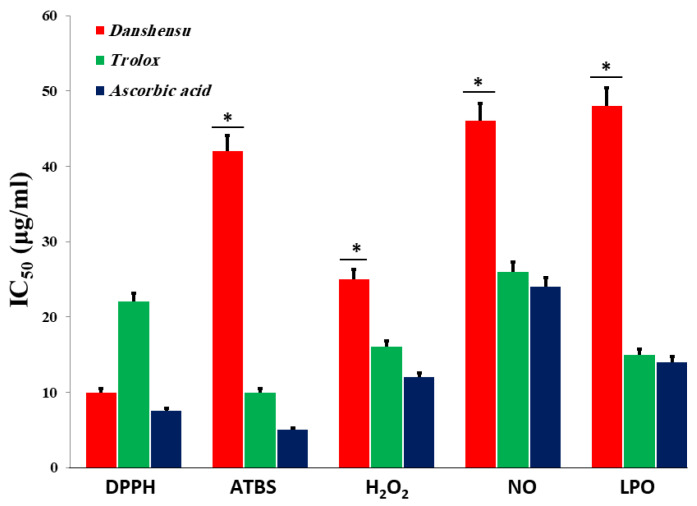
The IC_50_ (µg/mL) values of *Danshensu* extracts for scavenging activity by 1, 1-diphenyl-2-picrylhydrazyl (DPPH) activity, total reduction capability (TRC), ABTS radical cation decolorization (ABTS), hydrogen peroxide (H_2_O_2_), nitric oxide (NO), lipid peroxidation (LPO) assay (Lower IC_50_ value indicates higher antioxidant activity). Each point represents the mean ± SD (*n* = 3). * shows statistically significant differences at *p* < 0.05.

The DPPH radicals were scaled by reducing the absorbance at 517 nm induced by DSS, which has antioxidant activity [53]. As shown in Figure 2A, at a concentration of 160 μg/mL, the IC_50_ values of water extracts of *Danshensu*, trolox, and ascorbic acid were 10, 22.2, and 7.5 μg/mL, respectively. In the present study, the IC_50_ value of the *Danshensu* extract was demonstrated to have significantly higher free radical scavenging activity compared to the standard, trolox, while the lower IC_50_ value indicates a higher free radical scavenging activity (Figure 3).

#### 3.2.2. Total Reduction Capability

The reducing ability of the *Danshensu* extract was determined, and as shown in Figure 2B, the reducing ability of the *Danshensu* extract was increased. Also, the trolox and ascorbic acid concentrations increased. The absorbance of *Danshensu* at 160 μg/mL was 0.28, while trolox and ascorbic acid functioned as a positive control, and the reducing powers at 160 μg/mL were 0.25 and 0.30, respectively. The results indicated that *Danshensu* extract had a clear difference in ferric ion-reducing ability, as compared to ascorbic acid.

#### 3.2.3. ABTS Radical Cation Decolorization Assay

The results from the ABTS+ radical scavenging ability were found to be high in Danshensu (IC50 = 42.21 μg/mL), followed by ascorbic acid (IC50 = 10.06 ± 1.06 μg/mL) and trolox (5 ± 1.9 μg/mL) (Figure 3). *Danshensu*, ascorbic acid, and trolox exhibited dose-dependent effective antioxidant activity (Figure 2C). The result indicated the ability of DSS to scavenge excess radicals [54].

#### 3.2.4. Hydrogen Peroxide (H_2_O_2_) Radical Scavenging Activity

The scavenging abilities of *Danshensu*, ascorbic acid, and trolox are shown in Figure 2D. H_2_O_2_ radical scavenging abilities of extracts of *Danshensu*, ascorbic acid, and trolox at a concentration of 160 µg/mL were 54.3 ± 1.07, 58.6 ± 1.07, and 62.43 ± 1.4%, respectively. The IC_50_ values of *Danshensu*, ascorbic acid, and trolox were found to be 25.23 ± 2.1, 16 ± 2.51, and 12.43 ± 1.4 μg/mL, respectively. The IC_50_ value indicates that the plant extract is a better hydroxyl radical scavenger than the standard ascorbic acid and trolox (Figure 3). The hydroxyl radical scavenging activity was increased by the extract with increasing concentration. The lipid peroxidation data reflect the scavenging abilities of *Danshensu* against hydroxyl radicals [55].

#### 3.2.5. Nitric Oxide (NO) Radical Scavenging Activity

From the analysis, the water extract of *Danshensu* showed the highest inhibitory effect with an IC_50_ value of 46 ± 2.01 μg/mL at a concentration of 160 μg/mL. However, trolox and ascorbic acid have a good inhibitory effect, with an IC_50_ value of 26.53 ± 1.7 and 24.23 ± 3.2 µg/mL, respectively (Figure 3). Therefore, *Danshensu* has an effective scavenging performance, as compared with trolox and ascorbic acid, at a concentration of 160 μg/mL (Figure 2E). Previous research has articulated the activity of phenolic compounds to suppress NO radicals [56].

#### 3.2.6. Inhibition of Lipid Peroxidation (LPO)

Previous studies documented the activity of phytochemical herbals to inhibit LPO in a dose–concentration manner (Figure 2F) [46]. The inhibition at a concentration of 160 μg/mL is followed in the order *Danshensu* < trolox < ascorbic acid. At a concentration of 160 μg/mL, *Danshensu* showed the highest inhibition of LPO with 78 ± 2.21% (*p* < 0.05), whereas trolox showed 56.66 ± 1.45% of inhibitory effects. Ascorbic acid was also utilized as a positive control and significantly inhibited LPO by 85.93 ± 1.86%. The IC_50_ values of *Danshensu*, trolox, and ascorbic acid were recorded to be 48.13 ± 2.32, 15.21 ± 1.01, and 14.31 ± 1.03 µg/mL, respectively (Figure 3). The results corroborated the efficacy of *Danshensu* as a promising source for inhibiting LPO.

### 3.3. In Vivo Study

#### 3.3.1. Acute Toxicity of Danshensu on Animals

The result indicated that there is no mortality of rats after treatment with various doses up to 3000 mg/kg of *Danshensu for* 15 days, which signifies that there are no noticeable signs of toxicity in rats in this study.

#### 3.3.2. Danshensu Combined with PROBIOTICS—Effect on Body Weight

After the end of these experiments, the final body weight of all groups was slightly influenced when compared to their initial body weight. As Table 3 displays, the final body weights of all groups were 182, 225, 181, 202, 190, and 187 g for Group A, Group B, Group C, Group D, Group E, and Group F, respectively. The initial body weights of all the groups were 170.5, 185.2, 176.2, 175.3, 177.22, and 174.22 g for Group A, Group B, Group C, Group D, Group E, and Group F, respectively. The activity of Group E (*Danshensu* + *L. acidophillius* mixture) and Group F (high-cholesterol-fed (HCF) + *Danshensu* + *L. casei* mixture), according to the findings, helped control the rats’ body weight. Also, the comparison of the final body weights of the HCF + FF, HCF + DSSE, *L. acidophillius* at a concentration of 2 × 10^8^ CFU/mL mixture with *Danshensu* (50 mg/kg/day), and *L. casei* at a concentration of 2 × 10^8^ CFU/mL mixture with *Danshensu* (50 mg/kg/day), against the HCF groups indicated a remarkable reduction in the rats’ weight, as compared to the HCF group. This finding indicated that *Danshensu* extract associated with probiotics has a significant impact on the body’s metabolism.

#### 3.3.3. Biochemical Parameters

The results of the lipid profile (TC, TG, VLDL-C, and LDL-C) were significantly higher in group B compared to the control animal Group A, as shown in Table 4. Total cholesterol was reduced to 126, 112, 94, 81, and 77 mg/dL, respectively, in animals treated with *Danshensu* (50 mg/kg/day), *Danshensu* (50 mg/kg/day) + *L. acidophillius* mixture at a concentration of 2 × 10^8^ CFU/mL, and *Danshensu* (50 mg/kg/day) mixed with *L. casei* at a concentration of 2 × 10^8^ CFU/mL. *Danshensu* (50 mg/kg/day) alone, *Danshensu* (50 mg/kg/day) plus a *L. acidophillius* mixture at a concentration of 2 × 10^8^ CFU/mL, and *Danshensu* (50 mg/kg/day) mixed with *L. casei* at a concentration of 2 × 10^8^ CFU/mL all increased HDL-C levels to 41, 50, 56, and 61 mg/dL, respectively. When compared to the HCF group value of 33 mg/dL, there was a reduction in LDL-C levels to 89, 63, 56, and 52 mg/dL, respectively, when compared to the HCF group value of 141 mg/dL. On blood lipid parameters, the *Danshensu* extract was found to have comparable potencies to fenofibrate-treated animals. Previous studies have reported that the blood lipid profile level is a significant biomarker for hyperlipidemia [57]. In the present work, *Danshensu* also improved the lipid profile, which was quite comparable with other studies [58].

#### 3.3.4. Oxidative Stress Markers

In hyperlipidemic conditions, antioxidative enzymes, such as SOD and reduced GSH, are changed, potentially induced to generate reactive oxygen species (ROS), mediating the organ’s damage [49]. As shown in Table 5, the OS marker (SOD, GSH, TBARS, and NO) levels were evaluated as significant (*p* < 0.05) in the blood serum, heart, and liver of HCF rats when compared to the NC group, as shown in Table 3.

#### 3.3.5. Serum

As Table 5 displays, the SOD level decreased significantly in HCF animals (*p* < 0.01) by 56.34% compared with those in the NC group. The *Danshensu* treatment group magnificently increased the serum SOD level by 55.4%. However, the *Danshensu + L. acidophillius* mixture and *Danshensu + L. casei* mixture treatments did not make any significant changes in serum SOD parameters. Also, there was no detectable change in the other measured parameters.

#### 3.3.6. Heart

As Table 5 summarizes, rats in Group B, who were fed a high-fat diet, had considerably lower levels of the antioxidant markers SOD and GSH than rats in Group A (who were fed a regular diet), by 49.2 and 34.2%, respectively. SOD, an antioxidant marker, was considerably upregulated by 56.2, 51.3, and 51.1% in Groups D, E, and F, respectively. In Groups HCF + DSS, HCF + DSS + *L. acidophillius*, and HCF + DSS + *L. casei*, the antioxidant indicator GSH was also considerably upregulated by 35.2, 45.5, and 48.3%, respectively. The other measured values, however, showed no obvious variations.

#### 3.3.7. Liver

As Table 5 reveals, rats in Group B had considerably lower levels of the antioxidant indicators SOD and GSH, whereas animals in Group A (on a normal diet) had much greater levels of TBARS. In Groups HCF + DSS, HCF + DSS + *L. acidophillius*, and HCF + DSS + *L. casei*, the antioxidant marker SOD was considerably upregulated by 40.21, 48.1, and 50.12%, respectively. Similar to this, Groups HCF + DSS, HCF + DSS + *L. acidophillius*, and HCF + DSS + *L. casei* showed significantly higher levels of the antioxidant marker GSH by 23.3, 30.21, and 38.23%, respectively. The TBARS levels did, however, dramatically decline by 55.59%. The other measured parameters did not change significantly.

#### 3.3.8. Determination of HMG-CoA Reductase Activity

**Figure 4:**
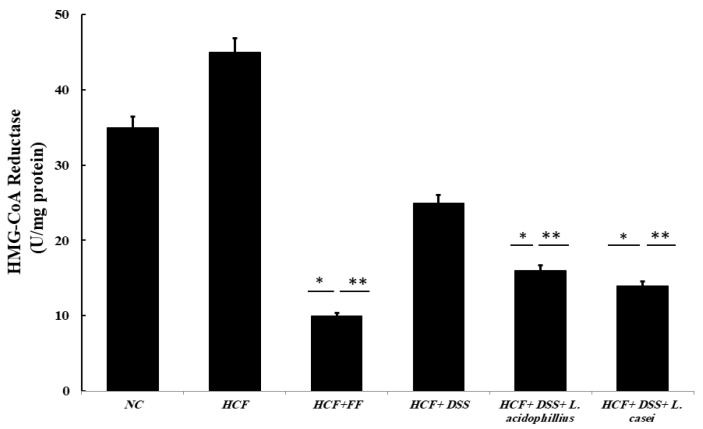
Determination of HMG-CoA reductase activity: NC (normal control): normal diet and water (control), HCF (high-cholesterol diet): normal diet + cholesterol (25 mg/kgb.w./day), HCF + FF: normal diet + cholesterol (25 mg/kgb.w./day) + fenofibrate (65 mg/kg b.w./day), HCF + DSS: normal diet + cholesterol (25 mg/kg b.w./day). *Danshensu* (50 mg/kg/day), HCF + DSS + *L. acidophillius*: normal diet + cholesterol (25 mg/kgb.w./day) + *L. acidophillius* mixture of 2 × 10^8^ CFU/mL plus *Danshensu* (50 mg/kg/day), HCF + DSS + *L. casei:* normal diet + cholesterol (25 mg/kgb.w./day) + *L. casei* mixture of 2 × 10^8^ CFU/mL plus *Danshensu* (50 mg/kg/day). * *p* < 0.05 vs. NC group, ** *p* < 0.01 vs. HCF group.

A statistically significant dose-dependent inhibitory effect of the *Danshensu* extract combined with probiotics on HMG-CoA reductase was observed when compared with the control. As shown in Figure 4, the HMG-CoA reductase was increased by 30% when compared to the normal control. The HMG-CoA reductase was decreased by 30%, 53%, and 60% for HCF + *Danshensu*, HCF + *Danshensu* + *L. acidophillius* mixture, and HCF + *Danshensu* + *L. casei* mixtures, respectively. Fenofibrate showed a larger reduction in the HMG-CoA reductase enzyme activity, at 73% (*p* < 0.001), as displayed in Figure 3.

#### 3.3.9. Determination of Hepatic and Fecal Lipids and Fecal Bile Acids

**Figure 5:**
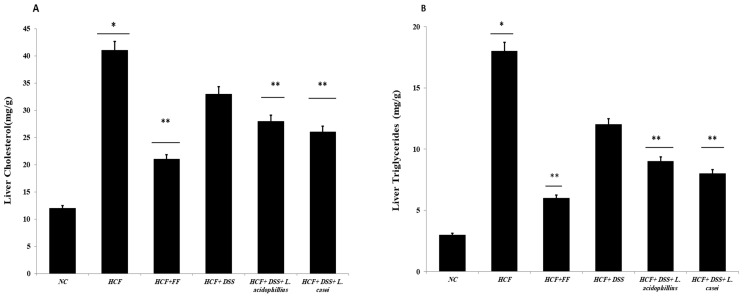
(**A**) Determination of liver cholesterol, (**B**): determination of liver triglycerides. NC (normal control): normal diet and water (control), HCF (high-cholesterol diet): normal diet + cholesterol (25 mg/kgb.w./day), HCF + FF: normal diet + cholesterol (25 mg/kgb.w./day) + fenofibrate (65 mg/kg b.w./day), HCF + DSS: normal diet + cholesterol (25 mg/kg b.w./day). *Danshensu* (50 mg/kg/day), HCF + DSS + *L. acidophillius*: normal diet + cholesterol (25 mg/kgb.w./day) + *L. acidophillius* mixture of 2 × 10^8^ CFU/mL plus *Danshensu* (50 mg/kg/day), HCF + DSS + *L. casei:* normal diet + cholesterol (25 mg/kgb.w./day) + *L. casei* mixture of 2 × 10^8^ CFU/mL plus *Danshensu* (50 mg/kg/day). * *p* < 0.05 vs. NC group, ** *p* < 0.01 vs. HCF group.

**Figure 6:**
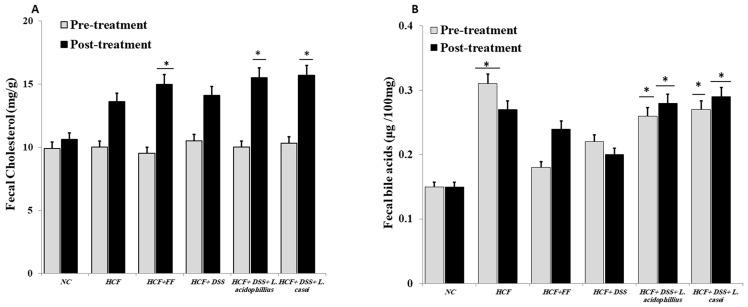
(**A**) Determination of fecal cholesterol, (**B**): determination of fecal bile acids: NC (normal control): normal diet and water (control), HCF (high-cholesterol diet): normal diet + cholesterol (25 mg/kgb.w./day), HCF + FF: normal diet + cholesterol (25 mg/kgb.w./day) + fenofibrate (65 mg/kg b.w./day), HCF + DSS: normal diet + cholesterol (25 mg/kg b.w./day). *Danshensu* (50 mg/kg/day), HCF + DSS + *L. acidophillius*: normal diet + cholesterol (25 mg/kgb.w./day) + *L. acidophillius* mixture of 2 × 10^8^ CFU/mL plus *Danshensu* (50 mg/kg/day), HCF + DSS + *L. casei:* normal diet + cholesterol (25 mg/kgb.w./day) + *L. casei* mixture of 2 × 10^8^ CFU/mL plus *Danshensu* (50 mg/kg/day). * *p* < 0.05 vs. NC group.

As seen in Figure 5A,B, the liver’s cholesterol and triglyceride levels were substantially less tightly controlled than those of the animals on a regular diet, following the ingestion of the probiotic-associated *Danshensu* extract. Similar to this, after giving the rats the hyperlipidemic drug, fenofibrate, the levels of cholesterol and triglycerides in the liver showed a substantially lower regulation. Additionally, the high-fat-diet animal group had greater fecal TC and bile acid levels than the animal group on a regular diet. When compared to the normal control group, the animal administration of *Danshensu* and probiotics resulted in a statistically significant rise in fecal TC levels, while animals treated with fenofibrate did not show any statistically significant increase in fecal TC or bile acid levels when compared with the hyperlipidemic control group (Figure 6A,B).

#### 3.3.10. Histopathological Changes

##### Liver

**Figure 7:**
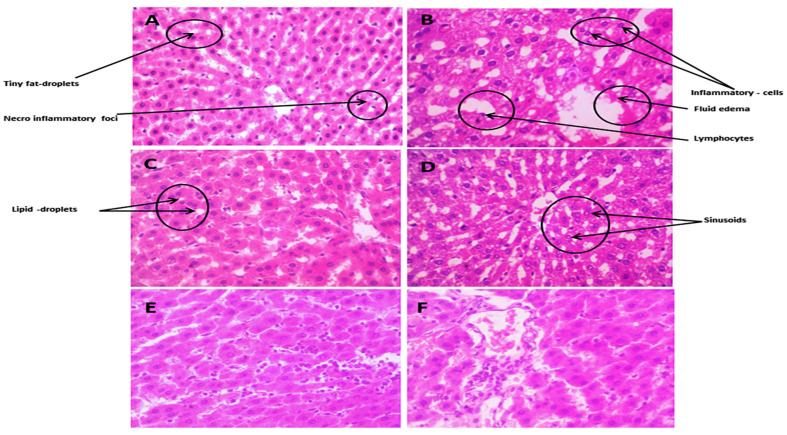
The pathological observation of the effect of *Danshensu* extracts in hepatic tissues: (**A**) normal liver with glomerulus. (**B**) High cholesterol-fed (HCF) hepatic tissue section with large area of necrosis, congested central vein (CV), and lipid droplets (LD) accumulation. (**C**) Fenofibrate-treated hepatic tissue showing restoration of hepatic structure. (**D**) *Danshensu* (50 mg/kg/day) treated liver; (**E**) *Danshensu* (50 mg/kg/day) mixed with *L. acidophillius* at concentration of 2 × 10^8^ CFU/mL treated liver; (**F**) *Danshensu* (50 mg/kg/day) mixed with *L. casei* at concentration of 2 × 10^8^ CFU/mL treated liver, depicting improvement in the hepatic structure and sufficient reduction in the appearance of LD.

The liver tissue section of the normal liver revealed normal hepatic texture lobules with glomerulus, which were made up of radiating plates or strands of polygonal cells, with prominent round nuclei and eosinophilic cytoplasm, vertical to the central vein. Sinusoids were lined by a discontinuous layer of fenestrated endothelial cells with a fine arrangement of Kupffer cells. The portal area revealed the normal histological structure of the bile duct, portal vein, and hepatic artery shown in Figure 7A. As the figure displays, an HCF liver featured a large area of necrosis, congested central vein (CV), and lipid droplets (LDs) accumulation. Furthermore, the hepatic parenchyma showed disorganization of the hepatic cords and necrobiotic changes in hepatocytes characterized by focal necrotic foci and the hydropic degeneration of hepatocytes. A small number of micro-vesicular steatosis and apoptotic bodies were displayed. As shown in Figure 7B, fenofibrate-treated livers restored hepatic structures, composed of cords of polygonal cells with prominent round nuclei and eosinophilic cytoplasm, just like the control group, as shown in Figure 7C. Figure 7D shows that the sizes of lipid droplets in the *Danshensu* group were remarkably smaller than those of the HCF group, suggesting that *Danshensu* combined with probiotics could reduce the accumulation of lipid droplets. Figure 7E,F show that the *Danshensu* combined with probiotics treatment group can maintain normal hepatocytes in HCF rats by preventing or reducing excess lipid formation.

##### Kidney

**Figure 8:**
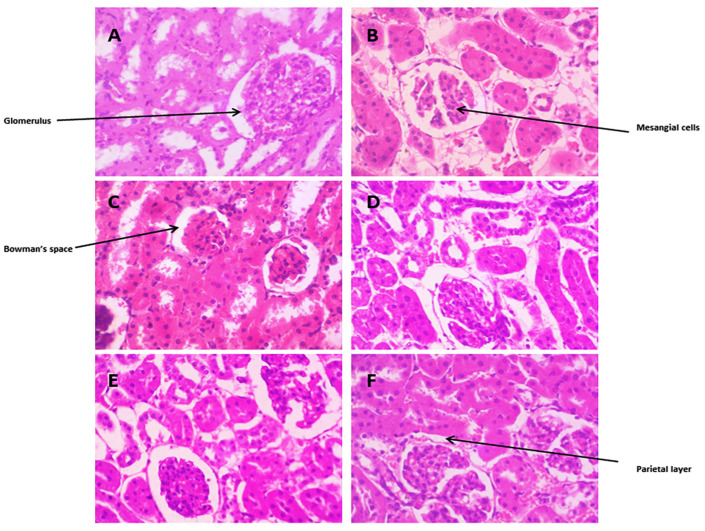
Histopathological effects of *Danshensu* extracts combined with probiotics in kidney of high-cholesterol-fed (HCF) rat. (**A**) Represents hematoxylin eosin staining of normal kidney with glomerulus. (**B**) High-cholesterol-fed (HCF) group revealed shrinkage of capillary tufts with widening of Bowman’s space of some glomeruli. (**C**) Fenofibrate-treated kidney tissue showing restoration of kidney structure. (**D**) *Danshensu* (50 mg/kg/day) treated kidney tissue; (**E**) *Danshensu* (50 mg/kg/day) mixed with *L. acidophillius* at concentration of 2 × 10^8^ CFU/mL treated kidney tissue; (**F**) *Danshensu* (50 mg/kg/day) mixed with *L. casei* at concentration of 2 × 10^8^ CFU/mL treated kidney tissue, depicting improvement in glomerular structure and restored the Bowman’s space of the glomerulus in kidney sections.

The kidney tissue section of normal kidney tissue displayed a normal histological structure, characterized by circumscribed glomeruli with a normal structure of capillary tufts and Bowman’s capsule. As Figure 8A shows, the renal tubules of both proximal and distal convoluted tubules showed an intact epithelial lining and regular arrangement. As Figure 8B displays, HCF rats showed the shrinkage of capillary tufts with widening of Bowman’s space of some glomeruli. Also, wider Bowman’s space, severe hemorrhages in glomeruli, mild intertubular hemorrhages, and mild dilatations were observed in Group B. As Figure 8C reveals, the fenofibrate-treated kidney tissue section revealed restoration of the bowman’s space of glomerulus and tubular epithelial cell degeneration, without significant necrosis or apoptosis. The glomeruli showed a mild degree of shrinkage of glomerular tufts. As shown in Figure 8D, *Danshensu* improved the glomerular structure. *Danshensu* combined with probiotics restored the Bowman’s space of the glomerulus in kidney sections, as displayed in Figure 8E,F.

##### Pancreatic

**Figure 9:**
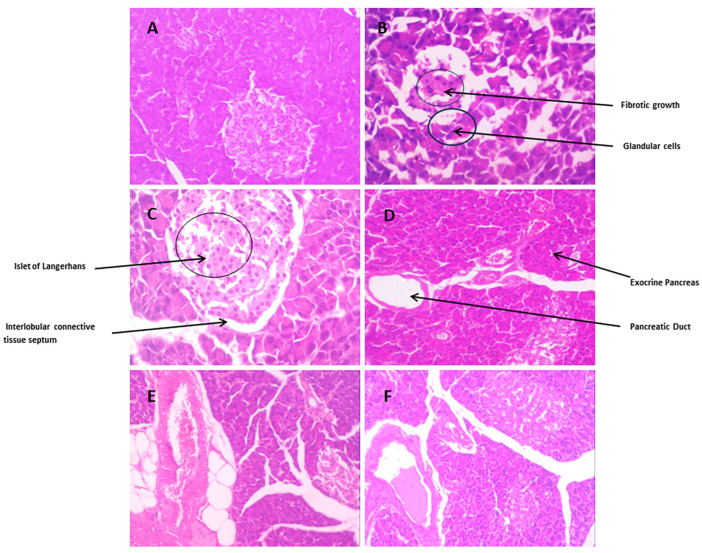
The pathological observation of the effect of *Danshensu* combine with probiotics in pancreatic: (**A**) normal pancreatic tissue. (**B**) High-cholesterol-fed (HCF) pancreatic tissue section with fibrosis in the b-cells. (**C**) Fenofibrate-treated pancreatic tissue displaying restoration of pancreatic structure. (**D**) *Danshensu* (50 mg/kg/day) treated liver; (**E**) *Danshensu* (50 mg/kg/day) mixed with *L. acidophillius* at concentration of 2 × 10^8^ CFU/mL treated normal pancreatic tissue; (**F**) *Danshensu* (50 mg/kg/day) mixed with *L. casei* at concentration of 2 × 10^8^ CFU/mL treated normal pancreatic tissue, depicting improvement in pancreatic structure and restoration to the normal pancreatic structure.

The pancreatic tissue section of normal pancreatic tissue displayed a normal histological structure of the pancreas with acinar cells, with a normal structure of both exocrine and endocrine tissues, as shown in Figure 9A. As Figure 9B displays, HCF rats revealed fibrosis in the b-cells of the pancreas. Figure 9C represents a fenofibrate-treated pancreatic section, showing reversal of the normal pancreatic structure. As Figure 9D reveals, the pancreatic tissue treated with *Danshensu* extract shows an improvement in the pancreatic structure. As Figure 9E,F displays, the pancreatic tissues restore the normal pancreatic structure, when treated with *Danshensu* combined with probiotics.

##### Coronary Blood Vessels

**Figure 10:**
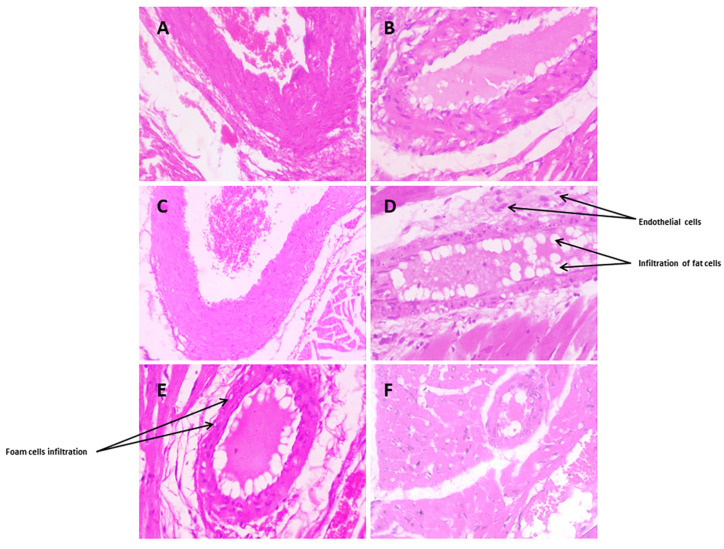
Histopathological effects of *Danshensu* combined with probiotics in coronary blood vessels of high-cholesterol-fed (HCF) rat (**A**) represents hematoxylin eosin staining of normal liver with glomerulus. (**B**) High-cholesterol-fed (HCF) liver with large area of necrosis, congested central vein (CV), and lipid droplets (LD) accumulation. (**C**) Fenofibrate-treated liver showing restoration of hepatic structure. (**D**) *Danshensu* (50 mg/kg/day) treated liver; (**E**) *Danshensu* (50 mg/kg/day) mixed with *L. acidophillius* at concentration of 2 × 10^8^ CFU/mL treated liver; (**F**) *Danshensu* (50 mg/kg/day) mixed with *L. casei* at concentration of 2 × 10^8^ CFU/mL treated, depicting that the coronary blood vessels revealed regular arrangement of endothelial lining.

Coronary arterial vessels of normal blood vessels displayed a normal histological structure with normal distinct layers of tunica intima, internal elastic lamina, tunica media (many layers of smooth muscle), and external elastic lamina (multiple layers), extending into the tunica adventitia, as Figure 10A shows. Figure 10B displays that HCF rats revealed damaged endothelial cells and the accumulation of fat in the form of foam cell infiltration, with a score of 3, with perivascular edema (intravascular edema). As Figure 10 shows, coronary arterial vessels were treated with fenofibrate; the coronary blood vessels were restored to normal vessels with a score of 1, characterized by minor severity with slight damage to endothelial cells. The coronary arterial vessels treated with *Danshensu* extract showed an improvement in the structure of coronary blood vessels, as outlined in Figure 10D. Figure 10E,F show that probiotics combined with the *Danshensu* extract affected the coronary blood vessels’ regular arrangement of endothelial lining with an accumulation of few fat droplets with a score of 1.

### 3.4. Quantitative RT-PCR

**Figure 11:**
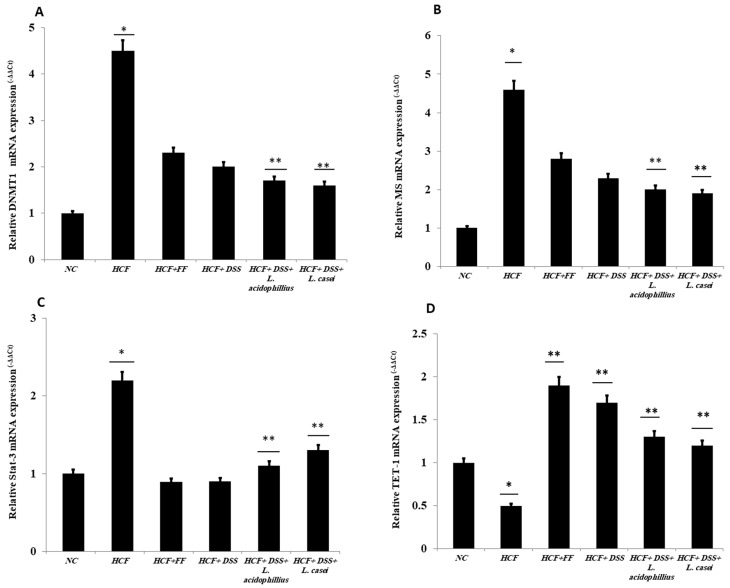
RT-PCR of *Danshensu* combined with probiotics. (**A**) DNMT1, (**B**) MS, (**C**) STAT-3, (**D**) TET-1. NC (normal control): normal diet and water (control), HCF (high-cholesterol diet): normal diet + cholesterol (25 mg/kgb.w./day), HCF + FF: normal diet + cholesterol (25 mg/kgb.w./day) + fenofibrate (65 mg/kg b.w./day), HCF + DSS: normal diet + cholesterol (25 mg/kg b.w./day). *Danshensu* (50 mg/kg/day), HCF + DSS + *L. acidophillius*: normal diet + cholesterol (25 mg/kgb.w./day) + *L. acidophillius* mixture of 2 × 10^8^ CFU/mL plus *Danshensu* (50 mg/kg/day), HCF + DSS + *L. casei:* normal diet + cholesterol (25 mg/kgb.w./day) + *L. casei* mixture of 2 × 10^8^ CFU/mL plus *Danshensu* (50 mg/kg/day). * *p* < 0.05 vs. NC group, ** *p* < 0.01 vs. HCF group.

The expression of DNMT1, which responds to DNA ion activity, and methionine synthase (MS), which improves the performance of methyl groups, was assessed. Figure 11A shows that Group 2, which represents the positive control, increased to 4.5-fold as compared to the negative control (Group 1). Meanwhile, Groups 3, 4, 5, and 6 decreased to half the value, as compared to the positive control (Group 2) with 53, 50, 47, and 45%, respectively. Similarly, the MS gene-expressed value increased more than five-fold when compared with the positive control (Figure 11B), whereas the other groups, which represent *Danshensu* alone and *Danshensu* associated with different probiotics (Groups 4, 5, and 6), were downregulated by 40, 50, and 55% when compared to the positive control. Interestingly, the activity of STAT-3 contributes to the IL-6/STAT3 pathway, which functionalizes as a hepatoprotection pathway after liver damage. Figure 11C shows the activity of *Danshensu* as a hepatic protective agent, working alone or combined with different probiotics. Groups 4, 5, and 6 showed downregulation to 25, 30, and 34% as compared to the positive control (Group 2), which increased to two-fold when compared to the negative control. The ten-eleven translocation 1 (TET1) plays a crucial role in the demethylation process. Figure 11D reveals the downregulation of the TET1 in the positive control (Group 2) to be 50% when compared to the negative control. However, treatment of *Danshensu* alone or associated with different probiotics increased the gene expression by 65, 75, and 78% in Groups 4, 5, and 6, respectively.

### 3.5. In Silico Study

#### 3.5.1. Molecular Docking

**Figure 12:**
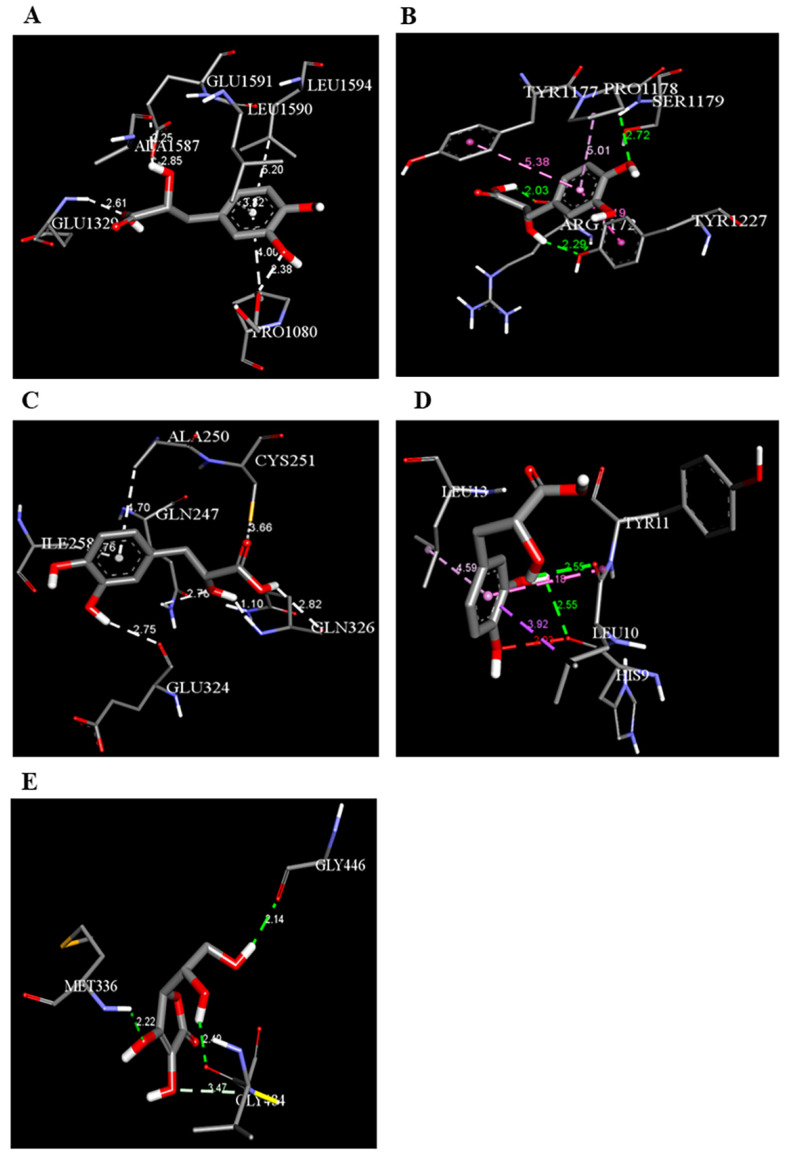
Three-dimensional structure of molecular docking between DSS and target proteins. (**A**) DNMT-1, (**B**) MS, (**C**) STAT-3, (**D**) TET-1, (**E**) HGM.

As Table 6 summarizes, DSS binds with DNAT-1 through hydrogen bond interactions with GLU 1329, ALA 1587, and EDO 1721. Additionally, it interacts with DNAT-1 with a pi–sigma bond (LEU 15910). Moreover, the DSS interacts with DNAT-1 via Pi-Alkyl (LEU 1594 and PRO1080); in contrast, there is an unfavorable acceptor–acceptor bond (GLU 1591). The binding energy of the interaction between DSS and DNAT-1 was −5.9 kcal/mol. Also, MS has an ΔG of −5.7 kcal/mol. Hydrogen bond residues were observed (ARG 1172, TYR1227 and, SER1179), and there are other bonds between DSS and MS: Pi-sigma (LEU1591), Pi-Alkyl (PRO1178), and Pi-Pi Stacked (TYR 1177). Interestingly, STAT-3 interacts with DSS via different bonds: hydrogen bond (GLN 247, GLN326 GLU 324, and CYS 251), Pi-sigma (LEU1591), Pi-Alkyl (ALA 250), and Pi-Pi Sigma (ILE 258); however, there is an unfavorable donor–donor bond (GLN 326), with the ΔG occurring due to the interaction between STAT-3 and DSS equal to −5.3 kcal/mol. Similarly, the binding energy of the interaction between DSS and TET-1 was −4.0 kcal/mol throughout various interaction bonds: van der Waals bond (CYS 14, LEU 12, TYR 12), hydrogen bond (HIS 9, LEU 10)), Pi-sigma (LEU10), Pi-Alkyl (LEU 13), and an unfavorable acceptor–acceptor bond (HIS 9). Finally, DSS was bound to HMG via a hydrogen bond (MET 336, VAL483 GLY 446) and carbon hydrogen bond (CLY 484), with the ΔG value at −5.0 kcal/mol. In summary, the docking results predict DSS as having a favorable binding to DNAT-1, which has a higher affinity than other proteins. The interaction between DSS and DNAT-1, as Figure 12A displays, shows that the bond length between DSS, GLU 1329, and ALA 1587 was 2.61 and 2.85 A°, respectively. The pi–sigma bond (LEU 15910) was 3.82 A°. Also, Pi-Alkyl (LEU 1594 and PRO1080) was 5.20 A° and 4.0 A°, respectively. Similarly, the interaction between DSS and MS, as Figure 12B demonstrates, displays the bond length between DSS, Arg 1172, and Ser 1179 was 2.29 A° and 2.72 A°, respectively. Also, interactions with TYR 1177 by Pi-Pi stacked with bond lengths of 5.1 A° and Pi-Alkyl (PRO1178) of 5.01 A° were recorded. Interestingly, the interaction between DSS and STAT-3, as Figure 12C displays, showed the bond length between DSS with GLN 247, GLN326 GLU 324 and CYS 251 was 2.76 A°, 2.82 and 3.66 A°, respectively. Also, the bond length between DSS ALA 250 and ILE 258 was 4.7 and 2.76 A°, respectively. The interaction between DSS and TET-1, as Figure 12D shows, indicates the bond length between DSS with HIS 9 and LEU 10 was 2.55 A° and 2.55 A°, respectively. Also, DSS interacts with LEU10 by Pi-sigma, with a bond length 3.92 A° and with LEU13 by Pi-Pi Pi-Alkyl bond, with a bond length of 4.59 A°. Finally, the interaction between DSS and HMG, as shown in Figure 11E, displayed that the bond length between DSS and MET 336, VAL483 and GLY 446 was 2.22, 2.42 and 2.14 A°, respectively. Finally, the bond length between DSS and CLY 484 was 3.47 A°.

#### 3.5.2. ADMET and Drug-Likeness Properties

To certify that the DSS can be utilized as feasible drugs, Table 7 summarizes the rule of Lipinski to determine the probability of DSS to be used as a drug. The molecular weight of DSS was 471.518 g/mol, and the number of hydrogen bond acceptors and hydrogen bond donors was 5 and 4, respectively. The synthetic accessibility of DSS is 1.91, which indicates that the DSS can be easily synthesized according to the scale that ranges from 1 (easier to synthesize) to 10 (very difficult to synthesize). The bioavailability of the DSS was 0.56, which designates compliance with the Lipinski rule of five, and the rat bioavailability value was 55%, which is compatible with the reference scale. Also, DSS is much safer according to their interactions with CYP families, which are responsible for the metabolism of DSS and include 1A2, 2C9, 2C19, 2D6, and 3A4. The table displays that DSS is a non-toxic agent. The absorption is 41.774. Finally, DSS showed satisfactory physicochemical and pharmacokinetic ADMET properties. Thus, on the basis of these outcomes, it can be acknowledged as a potential feasible drug (Table 8).

## 4. Discussion

Nonalcoholic fatty liver disease (NAFLD) is a major hepatic disorder occurring in non-alcohol-drinking individuals [1]. Nowadays, it is a global medical issue, and more than 25% of the general population worldwide suffers from NAFLD [59]. Epigenetic modifications combined with NAFLD are related to type 2 diabetes mellitus and other metabolic diseases [60,61]. Physiological changes in adolescence in short intervals contribute to dysmetabolism, which may lead to early liver damage [62]. Hyperlipoproteinemia is an abnormal overproduction of lipoproteins in the bloodstream due to a lack of dyslipidemia, which leads to a raised lipid level inside blood vessels and causes atherosclerosis [63]. Medicinal plants are the heart of traditional medicine, which are considered a valuable source of therapeutic agents for a wide range of diseases [64]. Nowadays, scientists are interested in medicinal plants’ antioxidant properties because of their superior effectiveness, safety, and consumer acceptability [65]. 3-(3, 4-dihydroxyphenyl)-(2*R*) lactic acid, or salvianic acid A (DSS), is derived from the root of Danshen (*Salvia miltiorrhiza*) [66]. Past articles have demonstrated the magnificent role of DSS as an anti-iron overload agent and a heart and liver protective agent [67,68,69]. The DSS mechanism depends upon antioxidation, anti-apoptosis, vasodilation, and inflammation regulation and lipidemia control occurring via signaling pathways, such as PI3K/Akt-ERK1/2/Nrf2/HO-1, Bcl-2/Bax, and eNOS [66]. Previous research has highlighted the role of *L. acidophilus* in the treatment of obesity, as well as a 3-week intake of probiotics (*L. acidophilus* SGL11, 1.5 × 10^10^ colony-forming units (CFUs)) capable of modulating body composition, gut bacterial composition, and psychopathological status in 60 obese and pre-obese women [70]. There are different classes of probiotics, including Firmicutes, Actinobacteria, Bacteroidetes, Proteobacteria, and Verrucomicrobia, but Bacteroidetes are the dominant strain. Probiotics, opportunistic pathogens, and pathogenic bacteria are the three types of GM. The balance between probiotics and pathogenic bacteria protects the intestinal mucosal barrier, improves metabolism and immunity, and helps the body intake nutrients, as well as resisting pathogenic microorganisms that attack the body [71]. Furthermore, the *Lactobacillus* strains have had a great effect on reducing obesity, insulin resistance, and pro-inflammatory responses [72]. The administration of a probiotic bacterium associated with *Danshensu* revealed an increase in hydroxyl radical removal activity in the current study. In this work, the *Danshensu* extracts combined with probiotics (*L. casei* and *L. acidophilus*) scavenged the NO, which reduced the chromophore formation and decreased the absorbance as the concentration of the plant extract increases. LPO production may lead to significant biological damage [63]. Scientists have shown that LPO is the main reason for oxidative stress (OS), leading to different diseases such as cardiovascular disease and cancer. Also, the generation of malondialdehyde (MDA) occurs through a set of chemical reactions [64]. In the present work, the LPO value was significantly suppressed by all the extracts in cooperation with *L. casei* and *L. acidophilus*. Thus, 50 mg/kg/day of *Danshensu* mixed with *L. casei* or *L. acidophillius* at a concentration of 2 × 10^8^ CFU/mL clearly corroborates the efficacy of *Danshensu* combined with probiotics as a promising source of LPO inhibition [73]. In the present work, the LPO value was significantly suppressed by all the extracts in cooperation with *L. casei* and *L. acidophilus*. Previous studies highlighted the greater role of the lyophilized *L. casei* IMVB-7280 as an anti-obesity agent than that of the lyophilized Bifidobacterium animalis VKB and VKL strains [74]. Interestingly, it reduces the Firmicutes to Bacteroidetes ratio and the expression of angiotensin-converting enzyme (ACE) to prevent metabolic-related hypertension [75]. Table 2 in this report demonstrates that elevated LDL-C and TC concentrations were accompanied by lower high-density lipoprotein levels, as compared to the process of atherosclerotic lesions [75]. In a previous report, it was mentioned that the high-fat-diet rats significantly increased the values of TC, LDL, VLDL, and TGs in the blood serum and also significantly decreased the level of HDL in the serum [76]. In this study, the *Danshensu* extract could be modified and improved to improve the lipid profile, which was quite comparable with another study [77]. Changes in oxidative stress parameters, such as SOD and reduced GSH, can cause ROS production, which leads to mediated damage [78]. As displayed in Table 3, the oxidative stress parameter (SOD, GSH, and NO) levels were significantly elevated (*p* < 0.05) in the serum, heart, and liver of high-fat-diet rats in comparison to those of the normal control group. Previous research also demonstrated that hypercholesterolemia suppresses the antioxidant defense system by reducing the activities of SOD and catalase in rats [78]. SOD and catalase enzymes have a significant role in the antioxidant defense mechanism [79]. Previous research has highlighted the role of *L. acidophilus* in the treatment of obesity, as well as a 3-week intake of probiotics (*L. acidophilus* SGL11, 1.5 × 10^10^ colony-forming units (CFUs)) capable of modulating body composition, gut bacterial composition, and psychopathological status in 60 obese and pre-obese women [80]. By studying HMG-CoA reductase, which controls and regulates the production and metabolism of cholesterol and lipids, we hope to gain a better understanding of how *Danshensu* extracts combined with probiotics work as antihyperlipidemic activity agents. Inhibition of the HMG-CoA reductase enzyme is the prime step in treating hyperlipidemia [81]. The data revealed an inhibitory effect of the *Danshensu* extract combined with probiotics on HMG-CoA reductase compared with the control. Also, the hepatic and fecal lipids and fecal bile acid levels in the hyperlipidemic control group showed a significant increase when compared with the normal control. Previous reports demonstrate that lipid drops are usually the result of aggregation in hepatic tissue, called hepatic steatosis, under the progress of atherosclerosis, especially at the hyperlipidemia stage. SOD and catalase enzymes, on the other hand, help to reduce excess lipid drops [76,79]. Also, the hepatic and fecal lipids and fecal bile acid levels in the hyperlipidemic control group showed a significant increase when compared with the normal control. Previous reports demonstrate that lipid drops are usually the result of aggregation in hepatic tissue, called hepatic steatosis, under the progress of atherosclerosis, especially in the hyperlipidemia stage. SOD and catalase enzymes, on the other hand, help to reduce excess lipid drops [74,75]. In our study, our data emphasized that treatment with the *Danshensu* extract keeps the hepatocytes in a normal state via a reduction in excess lipid formation in high-fat-diet rats. Previous studies indicated the crucial role of *Danshensu* in humans and animals in modulating liver injury, especially reversing liver fibrosis. It regulates the immunologic functions of hepatic NK cells during liver fibrosis. Interestingly, in addition to directly inhibiting HSC activation, promoting NK cell activity might also be one of the anti-fibrotic mechanisms [80]. Furthermore, histology of the kidney in high-cholesterol-fed (HCF) rat models revealed widening of Bowman’s space area, severe hemorrhages in glomeruli, intertubular hemorrhages, and mild dilatation, which could have resulted in the structural changes seen in the glomerulus [81], as shown in Figure 6B. These observed pathological changes were decreased by a combined probiotic associated with SME-treated HCF rats (Figure 8E,F). The glomeruli were revealed to be normal with mild hemorrhages because of the protective effect of *Danshensu. Danshensu* can decrease the GFR, which is responsible for the narrowing of the Bowman’s space in the glomerulus. The change in the structure of the rat’s pancreatic tissue cells was due to an elevation in lipid levels (Figure 8A). The function of pancreatic stellate cells is activated, which initiates the processes of free fatty acid (FFA) and lipid peroxide synthesis. It also aids in the process of pancreatic fibrogenesis [63]. Fibrosis is carried out in an extracellular matrix, in which the acinar cells disappear. The administration of *Danshensu* extract-associated probiotics decreased the progression of fibrosis, which contributes to protecting the pancreatic cells from damage (Figure 9E,F). Importantly, coronary artery disease is classified as the main reason for death. The overproduction of TG-rich lipoproteins in the liver may lead to hypertriglyceridemia, which is associated with decreased HDL-c and increased cholesterol levels [81]. The histological observations of the coronary artery vessels after treatment of HCF with the extract of *Danshensu* combined with a probiotic showed a regular arrangement of the endothelial lining with the accumulation of a few fat droplets (P0E–F). DNA methylation has a crucial role in the epigenetic process because it plays a significant role in multigenetic processes, like transcription and development. DNA methylation is defined as the transformation process of a methyl group onto the C5 position of 5′-CpG-3′ dinucleotides to form 5-methylcytosine (5 mC). The process is catalyzed by a specific enzyme called DNA methyltransferase (DNMT), while S-adenosyl methionine (SAM) works as an active methyl donor. The balance between DNA methyltransferases and demethylases is vital for genomic methylation homeostasis. Imbalances in genomic methylation homeostasis will cause many different diseases [82]. In our study, the activity of *Danshensu* extract combined with different probiotics decreased to half the value as compared to the positive control, as shown in the figure. Similarly, the methionine synthase MS gene showed that the activity of *Danshensu* alone and *Danshensu* associated with different probiotic Groups 4, 5, and 6 was downregulated by 40, 50, and 55%, respectively, when compared to the positive control. TET-1 is a hallmark of the demethylation process. The treatment of *Danshensu* alone or associated with different probiotics activates gene expression with 65, 75, and 78% in Groups 4, 5, and 6, respectively, when compared to the positive control (Group 2). The suppression of hepatic STAT 3 through *Danshensu* alone or in combination with different probiotics prevented NAFLD-induced liver fibrosis and diminished lipotoxicity [83]. Previous articles documented that the association between blueberries and probiotics (BP) can effectively dampen liver inflammation; hence, scientists recently targeted STAT3 to ameliorate fatty liver disease [83]. Also, STAT-3 acts as a critical anti-inflammatory signal to control NAFLD-induced liver inflammation and fibrosis [84]. The in silico study confirmed the activity of the DSS to interact with the DNMT-1, MS, STAT-3, TET-1, and HMG with hydrogen bond interaction with a good ΔG, equal to −5.9, −5.7, −5.3, −4.0, and −5.0 for DNMT-1, MS, STAT-3, TET1, and HMG, respectively. Interestingly, ADMET and drug-likeness properties certify that the *Danshensu* can be utilized as feasible drugs because *Danshensu* showed satisfactory physicochemical and pharmacokinetic ADMET properties. Finally, on the basis of these outcomes, it can be acknowledged that *Danshensu* has an ameliorative effect against methylation inhibitors in nonalcoholic fatty liver disease by decreasing the methylation activity genes. We recommend that *Danshensu* should be studied in depth, particularly the mechanism of restoration for the liver tissues’ texture and advanced study so that it can be applied as a novel drug.

## 5. Conclusions

In this study, we investigated the activity of *Danshensu* combined with different probiotics, such as *L. casei* and *L. acidophilus*, as an anti-methylation as well as antioxidant agent. We orally administered 2 × 10^8^ CFU/mL mixtures with *Danshensu* (50 mg/kg/day) to the rats after feeding them a high-cholesterol diet for 4 weeks. The results showed a significant reduction in the lipid blood test profiles as well as TBARS, which refers to the lipid peroxidation, and an increase in antioxidant markers, such as SOD, NO, and GSH. Also, it has an inhibitory effect on HMG-CoA reductase. The efficiency of *Danshensu* to block the methylation activity in DNMT-1, MS, TeT-1, and Stat-3 genes was outlined. The hepatic and fecal lipids and fecal bile acid levels were increased when compared with the normal control. The histopathological study and observation showed that the HCF- and *Danshensu*-treated group’s revealed a clear architecture in the liver, kidney, pancreas, and coronary blood vessels. Further studies are needed to study the bioactive compounds in depth and the elucidation of their molecular mechanisms.

## Figures and Tables

**Table 1:** List of primers using rt-PCR.

Primer	Sequence (5′-3′)
DNMT-1	F: AGGAATGTGTGAAGGAGAAATTG
	R: CTTGAACGCTTAGCCTCTCCATC
MS	F: AGAAGAGGATTATGGTGCTGGATG
	R: TCTTAATTCCTGTCTGGAGAGTT
STAT-3	F: ACCCAACAGCCGCCGTAG
	R: CAGACTGGTTGTTTCCATTCAGAT
TET1	F: ACTCCCTGAGGTCTGTCCTGGGA
	R: GGATCGAGACATAGCTACAGAGT
GAPDH	F: CAGGTTGTCTCCTGCGACTT
	R: TATGGG GGTCTGGGATGGAA

**Table 2:** Total phenolic content and total flavonoids of *Danshensu* extracts.

Parameter	*Danshensu* Extracts
Total Polyphenol Content (TPC)	111.9 ± 216 mg GAE g^−1^
Total flavonoids (TF)	33.79 ± 1.89 mg QE g^−1^

*n* = 3.

**Table 3:** Effect of *Danshensu* combined with probiotics (*Lactobacillus casei* and *Lactobacillus acidophilus*) on body weight for rats.

Group	Initial Body Weight-1st Day (g)	Final Body Weight-28th Day (g)
NC	170.5 ± 2.31	182 ± 2.8
HCF	185.2 ± 2.75	225 ± 3.22 *
HCF + FF	176.2 ± 3.93	181 ± 2.89
HCF + DSS	175.3 ± 3.43	202 ± 3.11 *
HCF + DSS + *L. acidophillius*	177.22 ± 2.31	190 ± 3.61
HCF + DSS + *L. casei*	174.22 ± 3.91	187 ± 2.3

NC (Normal control): normal diet and water (control), HCF (high-cholesterol diet): normal diet + cholesterol (25 mg/kgb.w./day), HCF + FF: normal diet + cholesterol (25 mg/kgb.w./day) + fenofibrate (65 mg/kgb.w./day), HCF + DSS: normal diet + cholesterol (25 mg/kgb.w./day). *Danshensu* (50 mg/kg/day), HCF + DSS + *L. acidophillius*: normal diet + cholesterol (25 mg/kgb.w./day) + *L. acidophillius* mixture of 2 × 10^8^ CFU/mL plus *Danshensu* (50 mg/kg/day), HCF + DSS + *L. casei:* normal diet + cholesterol (25 mg/kgb.w./day) + *L. casei* mixture of 2 × 10^8^ CFU/mL plus *Danshensu* (50 mg/kg/day).* *p* < 0.01 vs. Initial body weight, n = 6, mean ± SEM.

**Table 4:** The influence of *Danshensu* in combination with probiotic on male Wistar rats’ serum lipid profile.

Parameters (mg/dL)	NC	HCF	HCF + FF	HCF + DSS	HCF + DSS + *L. acidophillius*	HCF + DSS + *L. casei*
TC	73.04 ± 2.21	153.44 ± 3.21	126.12 ± 5.1 *	112 ± 3.7 *	94 ± 2.9	91 ± 1.67
TG	61.43 ± 2.31	121.21 ± 2.1	103.31 ± 3.97 *	94.18 ± 2.3	81.47 ± 2.1	77.31 ± 3.6
HDL	62.59 ± 3.62	33.54 ± 4.85	41.18 ± 2.1	50.45 ± 3.6	56.12 ± 3.4	61.11 ± 1.9
LDL	51 ± 2.33 **	141.32 ± 3.66	89.37 ± 5.9	63.17 ± 2.8	56.41 ± 1.6	52.17 ± 3.6
VLDL	15.86 ± 2.73	23.37 ± 3.97	20.47 ± 4.1	18.3 ± 1.09	16.8 ± 1.03 *	15.9 ± 0.97 *

NC (Normal control): normal diet and water (control), HCF (high-cholesterol diet): normal diet + cholesterol (25 mg/kgb.w./day), HCF + FF: normal diet + cholesterol (25 mg/kgb.w./day) + fenofibrate (65 mg/kg b.w./day), HCF + DSS: normal diet + cholesterol (25 mg/kg b.w./day). *Danshensu* (50 mg/kg/day), HCF + DSS + *L. acidophillius*: normal diet + cholesterol (25 mg/kgb.w./day) + *L. acidophillius* mixture of 2 × 10^8^ CFU/mL plus *Danshensu* (50 mg/kg/day), HCF + DSS + *L. casei:* normal diet + cholesterol (25 mg/kgb.w./day) + *L. casei* mixture of 2 × 10^8^ CFU/mL plus *Danshensu* (50 mg/kg/day).** *p* < 0.01 vs. Initial body weight, *n* = 6, mean ± SEM * *p* < 0.05 vs. NC group.

**Table 5:** The oxidative stress markers in serum, liver, and heart of the male Wistar rats.

Parameters		NC	HCF	HCF + FF	HCF + DSS	HCF + DSS + *L. acidophillius*	HCF + DSS + *L. casei*
Serum	SOD ^a^	39.1 ± 1.14	17.2 ± 2 ^++^	25 ± 3.6	29.6 ± 4.5	34.1 ± 3.4	38.6 ± 2.6 ^++^
	GSH ^b^	137 ± 4.5	203 ± 5.7	113 ± 3.1	121 ± 1.6	129 ± 4.3	135 ± 6.5
	TBARS ^c^	26.2 ± 1.1	41.8 ± 4.1	36.3 ± 3.81	31.23 ± 2.2	29.1 ± 1.8	27.3 ± 2.1
	NO ^d^	28.12 ± 1.32	51.4 ± 1.36	22.91 ± 1.51	36.23 ± 2.6	30.19 ± 1.23	25.31 ± 3.1
Liver	SOD ^a^	88 ± 1.3	51 ± 1.2 ^+^	69 ± 1.4	73 ± 1.2	79 ± 1.1	84 ± 1.3 **
	GSH ^b^	339 ± 8.6	265 ± 6.1	302 ± 5.7	309 ± 4.4	329 ± 5.7	343 ± 6.4
	TBARS ^c^	96.4 ± 5.2	161 ± 7.6 ^++^	142 ± 8.39	116 ± 5.6	104 ± 6.5	89.2 ± 7.4 **
	NO ^d^	21.3 ± 2.3	43.2 ± 2.1	27.1 ± 3.4	21.2 ± 2.1	16.1 ± 2.3	14.3 ± 0.6
Heart	SOD ^a^	69.4 ± 3.6	35.1 ± 1.4 ^+^	61.1 ± 2.5	62.2 ± 1.5	68.13 ± 0.98	69.1 ± 1.31 *
	GSH ^b^	123 ± 4.3	81 ± 3.4 ^++^	95 ± 4.3	101 ± 2.3 *	112 ± 5.8 **	117 ± 6.4 ***
	TBARS ^c^	36.2 ± 1.4	53.4 ± 4.3	41.1 ± 2.1	34.1 ± 3.2	30 ± 2.3	29 ± 2.1
	NO ^d^	15.1 ± 1.3	25.2 ± 1.4	21.16 ± 2.2	17.14 ± 1.6	15.10 ± 1.5	12.12 ± 1.1

NC (Normal control): normal diet and water (control), HCF (high-cholesterol diet): normal diet + cholesterol (25 mg/kgb.w./day), HCF + FF: normal diet + cholesterol (25 mg/kgb.w./day) + fenofibrate (65 mg/kg b.w./day), HCF + DSS: normal diet + cholesterol (25 mg/kg b.w./day). *Danshensu* (50 mg/kg/day), HCF + DSS + *L. acidophillius*: normal diet + cholesterol (25 mg/kgb.w./day) + *L. acidophillius* mixture of 2 × 10^8^ CFU/mL plus *Danshensu* (50 mg/kg/day), HCF + DSS + *L. casei:* normal diet + cholesterol (25 mg/kgb.w./day) + *L. casei* mixture of 2 × 10^8^ CFU/mL plus *Danshensu* (50 mg/kg/day). * *p* < 0.01 vs. Initial body weight, *n* = 6, mean ± SEM. ^a^: % Inhibition, ^b^: μg/mg, ^c^: μM/mg, ^d^: μM/mg. * *p* < 0.05 vs.HCF group, ** *p* < 0.01 vs. HCF group, *** *p* < 0.001 vs. HCF group, ^+^ *p* < 0.05 vs. NC group, ^++^ *p* < 0.01 vs. NC group. *n* = 6, mean ± SEM.

**Table 6:** Two-dimensional interactions of DSS with DNMT-1, MS, STAT-3, TET-1, and HGM proteins.

Compound	Protein	∆G (kcal/mol)	2D	Interactions
Danshensu	DNAT-1	5.9	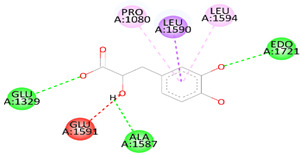	Hydrogen bond (GLU 1329, ALA 1587 and, EDO1721), Pi-sigma LEU1591), Pi-Alkyl (LEU 1594 and, PRO1080), unfavorable acceptor -acceptor bond (GLU 1591)
Danshensu	MS	−5.7	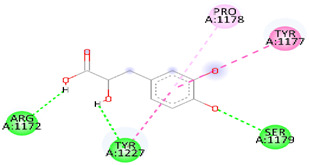	Hydrogen bond (ARG 1172, TYR1227 and, SER1179), Pi-sigma (LEU1591), Pi-Alkyl(PRO1178), and Pi-Pi Stacked (TYR 1177)
Danshensu	STAT-3	−5.3	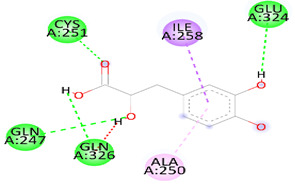	Hydrogen bond (GLN 247, GLN326 GLU 324 and, CYS 251), Pi-sigma (LEU1591), Pi-Alkyl(ALA 250), and Pi-Pi Sigma (ILE 258) Unfavorable Donor -Donor bond (GLN 326)
Danshensu	TET-1	−4.0	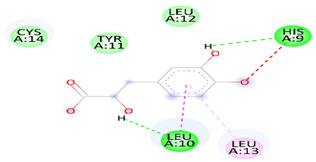	Van der Waals bond (CYS 14, LEU 12, TYR 12), Hydrogen bond (HIS 9, LEU 10)), Pi-sigma (LEU10), Pi-Alkyl (LEU 13) and, Unfavorable acceptor -acceptor bond (HIS 9)
Danshensu	HMG	−5.0	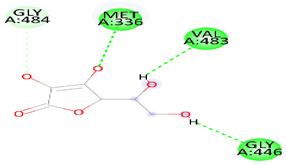	Hydrogen bond (MET 336, VAL483 GLY 446) and, Carbon Hydrogen Bond (CLY 484)

**Table 7:** Predicted drug-likeness properties of DSS.

Compound	Absorption Intestinal Human Absorption	Distribution		Metabolism								AMES Toxicity
		Log p	Log S	CYP2D6 Substrate	CYP3A4 Substrate	CYP1A2 Inhibitior	CYP2C19 Inhibitior	CYP2C9 Inhibitior	CYP2D6 Inhibitior	CYP3A4 Inhibitior	Total Clearance	
Danshensu	41.771	0.0858	−1.40	No	No	No	No	No	No	No	0.444	No

**Table 8:** Predicted ADMET properties of DSS.

Compound	Molecular Weight	HBA	HBD	mlogP	Synthetic Accessibility	Bioavailability	Lipinski Violation	Drug Likeness
Danshensu	470.518 g/mol	5	4	−0.04	1.91	0.56	0	Yes

## Data Availability

The original contributions presented in this study are included in the article Materials; further inquiries can be directed to the corresponding author.

## References

[1] Hyun J., Jung Y. (2020). DNA methylation in nonalcoholic fatty liver disease. Int. J. Mol. Sci..

[2] Del Campo J.A., Gallego-Durán R., Gallego P., Grande L. (2018). Genetic and epigenetic regulation in nonalcoholic fatty liver disease (NAFLD). Int. J. Mol. Sci..

[3] Pang Q., Zhang J.Y., Song S.D., Qu K., Xu X.S., Liu S.S., Liu C. (2015). Central obesity and nonalcoholic fatty liver disease risk after adjusting for body mass index. World J. Gastroenterol..

[4] Fitzpatrick E., Dhawan A. (2019). Childhood and adolescent nonalcoholic fatty liver disease: Is it different from adults?. J. Clin. Exp. Hepatol..

[5] Slomko H., Heo H.J., Einstein F.H. (2012). Minireview: Epigenetics of obesity and diabetes in humans. Endocrinology.

[6] Robertson K.D. (2005). DNA methylation and human disease. Nat. Rev. Genet..

[7] Illingworth R.S., Bird A.P. (2009). CpG islands—‘A rough guide’. FEBS Lett..

[8] Reik W., Dean W. (2001). DNA methylation and mammalian epigenetics. Electrophoresis.

[9] Guo J.U., Su Y., Zhong C., Ming G.L., Song H. (2011). Hydroxylation of 5-methylcytosine by TET1 promotes active DNA demethylation in the adult brain. Cell.

[10] Ito S., Shen L., Dai Q., Wu S.C., Collins L.B., Swenberg J.A., He C., Zhang Y. (2011). Tet proteins can convert 5-methylcytosine to 5-formylcytosine and 5-carboxylcytosine. Science.

[11] Jiao J., Sanchez J.I., Saldarriaga O.A., Solis L.M., Tweardy D.J., Maru D.M., Stevenson H.L., Beretta L. (2023). Spatial molecular and cellular determinants of STAT3 activation in liver fibrosis progression in non-alcoholic fatty liver disease. JHEP Rep..

[12] Mendoza J., Purchal M., Yamada K., Koutmos M. (2023). Structure of full-length cobalamin-dependent methionine synthase and cofactor loading captured in crystallo. bioRxiv.

[13] Hernández N.E., Tereschuk M.L., Abdala L.R. (2000). Antimicrobial activity of flavonoids in medicinal plants from Tafı del Valle (Tucuman, Argentina). J. Ethnopharmacol..

[14] Polya G. (2003). Biochemical Targets of Plant Bioactive Compounds: A Pharmacological Reference Guide to Sites of Action and Biological Effects.

[15] Maruthanila V.L., Poornima J., Mirunalini S. (2014). Attenuation of carcinogenesis and the mechanism underlying by the influence of indole-3-carbinol and its metabolite 3, 3′-diindolylmethane: A therapeutic marvel. Adv. Pharmacol. Pharm. Sci..

[16] Gorinstein S., Yamamoto K., Katrich E., Leontowicz H., Lojek A., Leontowicz M., Cíz M., Goshev I., Shalev U., Trakhtenberg S. (2003). Antioxidative properties of Jaffa sweeties and grapefruit and their influence on lipid metabolism and plasma antioxidative potential in rats. Biosci. Biotechnol. Biochem..

[17] Bao X.Y., Zheng Q., Tong Q., Zhu P.C., Zhuang Z., Zheng G.Q., Wang Y. (2018). Danshensu for myocardial ischemic injury: Preclinical evidence and novel methodology of quality assessment tool. Front. Pharmacol..

[18] Pang H., Wu L., Tang Y., Zhou G., Qu C., Duan J.A. (2016). Chemical analysis of the herbal medicine Salviae miltiorrhizae Radix et Rhizoma (Danshen). Molecules.

[19] Jovanović Stojanov S., Ntungwe E.N., Dinić J., Podolski-Renić A., Pajović M., Rijo P., Pešić M. (2023). Coleon U, Isolated from *Plectranthus mutabilis* Codd., Decreases P-Glycoprotein Activity Due to Mitochondrial Inhibition. Pharmaceutics.

[20] Won G., Choi S.I., Park N., Kim J.E., Kang C.H., Kim G.H. (2021). In vitro antidiabetic, antioxidant activity, and probiotic activities of Lactiplantibacillus plantarum and Lacticaseibacillus paracasei strains. Curr. Microbiol..

[21] Wegh C.A., Geerlings S.Y., Knol J., Roeselers G., Belzer C. (2019). Postbiotics and their potential applications in early life nutrition and beyond. Int. J. Mol. Sci..

[22] Masood M.I., Qadir M.I., Shirazi J.H., Khan I.U. (2011). Beneficial effects of lactic acid bacteria on human beings. Crit. Rev. Microbiol..

[23] Hadi A., Sepandi M., Marx W., Moradi S., Parastouei K. (2019). Clinical and psychological responses to synbiotic supplementation in obese or overweight adults: A randomized clinical trial. Complement. Ther. Med..

[24] Cakir M., Isbilen A., Eyupoglu I., Sag E., ÖREM A., Sen T., Kaklikkaya N., Kaya G. (2017). Effects of long-term synbiotic supplementation in addition to lifestyle changes in children with obesity-related non-alcoholic fatty liver disease. Turk. J. Gastroenterol..

[25] Hsu C.N., Hou C.Y., Chan J.Y., Lee C.T., Tain Y.L. (2019). Hypertension programmed by perinatal high-fat diet: Effect of maternal gut microbiota-targeted therapy. Nutrients.

[26] Cheng T.Y., Li J.X., Chen J.Y., Chen P.Y., Ma L.R., Zhang G.L., Yan P.Y. (2021). Gut microbiota: A potential target for traditional Chinese medicine intervention in coronary heart disease. Chin. Med..

[27] Nsimba R.Y., Kikuzaki H., Konishi Y. (2008). Antioxidant activity of various extracts and fractions of Chenopodium quinoa and Amaranthus spp. seeds. Food Chem..

[28] Zhishen J., Mengcheng T., Jianming W. (1999). The determination of flavonoid contents in mulberry and their scavenging effects on superoxide radicals. Food Chem..

[29] Brand-Williams W., Cuvelier M.E., Berset C. (1995). Use of a free radical method to evaluate antioxidant activity. LWT-Food Sci. Technol..

[30] Kumari S., Deori M., Elancheran R., Kotoky J., Devi R. (2016). In vitro and in vivo antioxidant, anti-hyperlipidemic properties and chemical characterization of *Centella asiatica* (L.) extract. Front. Pharmacol..

[31] Re R., Pellegrini N., Proteggente A., Pannala A., Yang M., Rice-Evans C. (1999). Antioxidant activity applying an improved ABTS radical cation decolorization assay. Free Radic. Biol. Med..

[32] Ruch R.J., Cheng S.J., Klaunig J.E. (1989). Prevention of cytotoxicity and inhibition of intercellular communication by antioxidant catechins isolated from Chinese green tea. Carcinogenesis.

[33] Sreejayan X.X., Rao M.N.A. (1997). Nitric oxide scavenging by curcuminoids. J. Pharm. Pharmacol..

[34] Ohkawa H., Ohishi N., Yagi K. (1979). Assay for lipid peroxides in animal tissues by thiobarbituric acid reaction. Anal. Biochem..

[35] Hassan A., Elebeedy D., Matar E.R., Elsayed A.F.M., Abd E.L., Maksoud A.I. (2021). Investigation of Angiogenesis and Wound Healing Potential Mechanisms of Zinc Oxide Nanorods. Front. Pharmacol..

[36] Hassan A., Al-Salmi F.A., Abuamara T.M.M., Matar E.R., Amer M.E., Fayed E.M.M., Hablas M.G.A., Mohammed T.S., Ali H.E., EL-fattah F.M.A. (2022). Ultrastructural analysis of zinc oxide nanospheres enhances anti-tumor efficacy against Hepatoma. Front. Oncol..

[37] Feng J., Fitz Y., Li Y., Fernandez M., Puch I.C., Wang D., Solomon S.B. (2015). Catheterization of the carotid artery and jugular vein to perform hemodynamic measures, infusions and blood sampling in a conscious rat model. JoVE (J. Vis. Exp.).

[38] Friedwald’s W.T., Levy I.R., Frederickson S.D. (1972). Estimation of concentration of low density lipoprotein cholesterol in plasma without use of the preparative ultracentrifuge. Clin. Chem..

[39] Ellman G.L. (1959). Tissue sulfhydryl groups. Arch. Biochem. Biophys..

[40] Marklund S., Marklund G. (1974). Involvement of superoxide anion radical in auto oxidation of pyrogallol and a convenient assay for superoxide dismutase. Eur. J. Biochem..

[41] Green L.C., Wagner D.A., Glogowski J., Skipper P.L., Wishnok J.S., Tannenbaum S.R. (1982). Analysis of nitrate, nitrite, and [15N] nitrate in biological fluids. Anal. Biochem..

[42] Hassan A., Al-Salmi F.A., Saleh M.A., Sabatier J.M., Alatawi F.A., Alenezi M.A., Albalwe F.M., Meteq R., Albalawi H., Darwish D.B.E. (2023). Inhibition Mechanism of Methicillin-Resistant Staphylococcus aureus by Zinc Oxide Nanorods via Suppresses Penicillin-Binding Protein 2a. ACS Omega.

[43] Thomsen R., Christensen M.H. (2006). MolDock: A new technique for high-accuracy molecular docking. J. Med. Chem..

[44] Hanwell M.D., Curtis D.E., Lonie D.C., Vandermeersch T., Zurek E., Hutchison G.R. (2012). Avogadro: An advanced semantic chemical editor, visualization, and analysis platform. J. Cheminform..

[45] Morris G.M., Huey R., Lindstrom W., Sanner M.F., Belew R.K., Goodsell D.S., Olson A.J. (2009). AutoDock4 and AutoDockTools4: Automated docking with selective receptor flexibility. J. Comput. Chem..

[46] Trott O., Olson A.J. (2010). AutoDock Vina: Improving the speed and accuracy of docking with a new scoring function, efficient optimization, and multithreading. J. Comput. Chem..

[47] Pires D.E., Blundell T.L., Ascher D.B. (2015). pkCSM: Predicting small-molecule pharmacokinetic and toxicity properties using graph-based signatures. J. Med. Chem..

[48] Daina A., Michielin O., Zoete V. (2017). SwissADME: A free web tool to evaluate pharmacokinetics, drug-likeness and medicinal chemistry friendliness of small molecules. Sci. Rep..

[49] Lipinski C.A., Lombardo F., Dominy B.W., Feeney P.J. (2012). Experimental and computational approaches to estimate solubility and permeability in drug discovery and development settings. Adv. Drug Deliv. Rev..

[50] Wang M., Li J., Rangarajan M., Shao Y., LaVoie E.J., Huang T.C., Ho C.T. (1998). Antioxidative phenolic compounds from sage (*Salvia officinalis*). J. Agric. Food Chem..

[51] Sharaf E.M., Hassan A., Al-Salmi F.A., Albalwe F.M., Albalawi H.M.R., Darwish D.B., Fayad E. (2022). Synergistic antibacterial activity of compact silver/magnetite core-shell nanoparticles core shell against Gram-negative foodborne pathogens. Front. Microbiol..

[52] Khella K.F., El Maksoud A.I.A., Hassan A., Abdel-Ghany S.E., Elsanhoty R.M., Aladhadh M.A., Abdel-Hakeem M.A. (2022). Carnosic acid encapsulated in albumin nanoparticles induces apoptosis in breast and colorectal cancer cells. Molecules.

[53] Ozsoy N., Can A., Yanardag R., Akev N. (2008). Antioxidant activity of Smilax excelsa L. leaf extracts. Food Chem..

[54] Cândido T.M., Ariede M.B., Pinto C.A.S.d.O., Lima F.V., Magalhães W.V., Pedro N.M.E., Padovani G., Sufi B.d.S., Rijo P., Velasco M.V.R. (2022). Rosmarinic Acid Multifunctional Sunscreen: Comet Assay and In Vivo Establishment of Cutaneous Attributes. Cosmetics.

[55] Liu C., Yu J., Zhang X. (2005). On changes of activity of antioxidases in hippocampus of rats with multi-infarct dementia and the intervention effects of acupuncture. China J. Tradit. Chin. Med. Pharm..

[56] Kumari S., Elancheran R., Kotoky J., Devi R. (2016). Rapid screening and identification of phenolic antioxidants *in Hydrocotyle sibthorpioides* Lam. by UPLC–ESI-MS/MS. Food Chem..

[57] Petitjean S.J., Lecocq M., Lelong C., Denis R., Defrère S., Mariage P.A., Alsteens D., Pilette C. (2022). *Salvia miltiorrhiza* Bunge as a potential natural compound against COVID-19. Cells.

[58] Ren J., Fu L., Nile S.H., Zhang J., Kai G. (2019). *Salvia miltiorrhiza* in treating cardiovascular diseases: A review on its pharmacological and clinical applications. Front. Pharmacol..

[59] Younossi Z.M., Koenig A.B., Abdelatif D., Fazel Y., Henry L., Wymer M. (2016). Global epidemiology of nonalcoholic fatty liver disease—Meta-analytic assessment of prevalence, incidence, and outcomes. Hepatology.

[60] Danford C.J., Yao Z., Jiang Z.G. (2018). Non-alcoholic fatty liver disease: A narrative review of genetics. J. Biomed. Res..

[61] Melton P.E., Burton M.A., Lillycrop K.A., Godfrey K.M., Rauschert S., Anderson D., Burdge G.C., Mori T.A., Beilin L.J., Ayonrinde O.T. (2023). Differential DNA methylation of steatosis and non-alcoholic fatty liver disease in adolescence. Hepatol. Int..

[62] Matias D., Nicolai M., Fernandes A.S., Saraiva N., Almeida J., Saraiva L., Faustino C., Díaz-Lanza A.M., Reis C.P., Rijo P. (2019). Comparison Study of Different Extracts of *Plectranthus madagascariensis*, *P. neochilus* and the Rare *P. porcatus* (Lamiaceae): Chemical Characterization, Antioxidant, Antimicrobial and Cytotoxic Activities. Biomolecules.

[63] Ntungwe E., Domínguez-Martín E.M., Bangay G., Garcia C., Guerreiro I., Colombo E., Saraiva L., Díaz-Lanza A.M., Rosatella A., Alves M.M. (2021). Self-Assembly Nanoparticles of Natural Bioactive Abietane Diterpenes. Int. J. Mol. Sci..

[64] Ntungwe E., Domínguez-Martín E.M., Teodósio C., Teixidó-Trujillo S., Armas Capote N., Saraiva L., Díaz-Lanza A.M., Duarte N., Rijo P. (2021). Preliminary Biological Activity Screening of *Plectranthus* spp. Extracts for the Search of Anticancer Lead Molecules. Pharmaceuticals.

[65] Nicolai M., Mota J., Fernandes A.S., Pereira F., Pereira P., Reis C.P., Robles Velasco M.V., Baby A.R., Rosado C., Rijo P. (2020). Assessment of the Potential Skin Application of *Plectranthus ecklonii* Benth. Pharmaceuticals.

[66] (2020). Potential Mechanisms Underlying the Hepatic–Protective Effects of Danshensu on Iron Overload Mice. Biol. Pharm. Bull..

[67] Gao Y., Wang N., Zhang Y., Ma Z., Guan P., Ma J., Zhang Y., Zhang X., Wang J., Zhang J. (2013). Mechanism of protective effects of Danshen against iron overload-induced injury in mice. J. Ethnopharmacol..

[68] Zhang Y., Zhang Y., Xie Y., Gao Y., Ma J., Yuan J., Li J., Wang J., Li L., Zhang J. (2013). Multitargeted inhibition of hepatic fibrosis in chronic iron-overloaded mice by *Salvia miltiorrhiza*. J. Ethnopharmacol..

[69] Aigner E., Weiss G., Datz C. (2015). Dysregulation of iron and copper homeostasis in nonalcoholic fatty liver. World J. Hepatol..

[70] Sitarek P., Synowiec E., Kowalczyk T., Bangay G., Śliwiński T., Picot L., Princiotto S., Rijo P. (2022). Anticancer Properties of *Plectranthus ornatus*-Derived Phytochemicals Inducing Apoptosis via Mitochondrial Pathway. Int. J. Mol. Sci..

[71] Farouk F., Zarka M.A., Al-Sawahli M.M., Hassan A., Mohamed A.F., Ibrahim I.M., Mohammed F.A.E.-R., Shebl R.I. (2023). Rosmarinic acid inhibits Rift Valley fever virus: In vitro, computational and analytical studies. Future Virol..

[72] Domínguez-Martín E.M., Magalhães M., Díaz-Lanza A.M., Marques M.P., Princiotto S., Gómez A.M., Efferth T., Cabral C., Rijo P. (2022). Phytochemical Study and Antiglioblastoma Activity Assessment of *Plectranthus hadiensis* (Forssk.) Schweinf. ex Sprenger var. hadiensis Stems. Molecules.

[73] Devi R., Sharma D. (2004). Hypolipidemic effect of different extracts of *Clerodendron colebrookianum* Walp in normal and high-fat diet fed rats. J. Ethnopharmacol..

[74] Stein O., Stein Y. (1999). Atheroprotective mechanisms of HDL. Atherosclerosis.

[75] Ahmed S.A., Hanif S., Iftkhar T. (2013). Phytochemical profiling with antioxidant and antimicrobial screening of *Amaranthus viridis* L. leaf and seed extracts. Open J. Med. Microbiol..

[76] Pal R., Girhepunje K., Shrivastav N., Hussain M.M., Thirumoorthy N. (2011). Antioxidant and free radical scavenging activity of ethanolic extract of Morinda citrifolia. Ann. Biol. Res..

[77] Deori M., Boruah D.C., Devi D., Devi R. (2014). Antioxidant and antigenotoxic effects of pupae of the muga silkworm *Antheraea assamensis*. Food Biosci..

[78] Wang H., Gao X.D., Zhou G.C., Cai L., Yao W.B. (2008). In vitro and in vivo antioxidant activity of aqueous extract from *Choerospondias axillaris* fruit. Food Chem..

[79] Bajpai V.K., Sharma A., Kang S.C., Baek K.H. (2014). Antioxidant, lipid peroxidation inhibition and free radical scavenging efficacy of a diterpenoid compound sugiol isolated from *Metasequoia glyptostroboides*. Asian Pac. J. Trop. Med..

[80] Chidambaram U., Pachamuthu V., Natarajan S., Elango B., Ramkumar K.M. (2013). In vitro evaluation of free radical scavenging activity of *Codariocalyx motorius* root extract. Asian Pac. J. Trop. Med..

[81] Bode J.G., Albrecht U., Häussinger D., Heinrich P.C., Schaper F. (2012). Hepatic acute phase proteins–regulation by IL-6-and IL-1-type cytokines involving STAT3 and its crosstalk with NF-κB-dependent signaling. Eur. J. Cell Biol..

[82] Zhao J., Qi Y.F., Yu Y.R. (2021). STAT3: A key regulator in liver fibrosis. Ann. Hepatol..

[83] Seo H.Y., Kim M.K., Lee S.H., Hwang J.S., Park K.G., Jang B.K. (2018). Kahweol ameliorates the liver inflammation through the inhibition of NF-κB and STAT3 activation in primary Kupffer cells and primary hepatocytes. Nutrients.

[84] Khalil H., Nada A.H., Mahrous H., Hassan A., Rijo P., Ibrahim I.A., Moahmed D.D., AL-Salmi F.A., Mohamed D.D., Elmaksoud A.I.A. (2024). Amelioration effect of 18β-Glycyrrhetinic acid on methylation inhibitors in hepatocarcinogenesis -induced by diethylnitrosamine. Front. Immunol..

